# Ion-Channel-Mediated Drug Repurposing Opportunities Validated by Single-Cell Perturbation in Colorectal Cancer

**DOI:** 10.3390/ijms27083412

**Published:** 2026-04-10

**Authors:** Zhongyuan Dong, Xuanlin Meng, Lianghua Wang

**Affiliations:** 1 Department of Biochemistry and Molecular Biology, College of Basic Medical Sciences, Naval Medical University, Shanghai 200433, China; zydongsmmu@126.com; 2 Key Laboratory of Biosafety Defense, Naval Medical University, Ministry of Education, Shanghai 200433, China

**Keywords:** drug repurposing, colorectal cancer, network pharmacology, ion channels, WGCNA, single-cell perturbation, protein–protein interactions, systems biology

## Abstract

Colorectal cancer (CRC) remains a leading cause of cancer mortality, yet no systematic effort has linked druggable CRC driver genes to downstream ion channel effectors. We integrated differential expression analysis, weighted gene co-expression network analysis (WGCNA), and protein–protein interaction (PPI) network pharmacology to identify CRC hub genes and their ion channel connections, validated by dual single-cell perturbation approaches: variational graph autoencoder-based virtual knockout (VGAE-KO) and experimental HCT116 CRISPRi Perturb-seq (6 genes, 8445 cells). WGCNA identified 100 hub genes spanning three functional programs. Ribosomal proteins link to K^+^ channels (*RPS21* → *KCNQ2*, targetable by EMA-approved ataluren, passed dual validation at 97.8th–98.7th percentile). RNA processing genes connect to Cl^−^ channels (*LSM7* → *CLIC1*, strongest signal at 99.8th–99.4th percentile). Immune checkpoint receptors (*LAG3*, *CD27*) connect via PPI intermediates to ${\mathrm{Ca}}^{2+}$ and K^+^ channels, targetable by relatlimab (FDA-approved) and varlilumab (Phase 2). This work maps previously unknown links between CRC driver genes and ion channel regulation, with the ataluren-*RPS21*-*KCNQ2* axis ready for pharmacological testing.

## 1. Introduction

Colorectal cancer (CRC) ranks third in cancer incidence and second in mortality worldwide, with 1.9 million new cases and 935,000 deaths annually [1]. CRC develops through well-characterized molecular pathways, including the adenoma–carcinoma sequence driven by APC/WNT dysregulation, the microsatellite instability (MSI) pathway involving mismatch repair deficiency, and the serrated neoplasia pathway [2,3]. Diagnosis relies on colonoscopy with histopathological confirmation, while staging follows the TNM system to guide treatment decisions [2]. Despite surgical advances and targeted therapies, systemic CRC treatment still relies on fluoropyrimidine-based chemotherapy (5-fluorouracil), typically combined with oxaliplatin or irinotecan [4]. Molecularly targeted agents (bevacizumab, cetuximab) and immune checkpoint inhibitors (pembrolizumab for MSI-H tumors) have improved outcomes in selected populations, yet these regimens produce significant toxicity, acquired resistance, and limited efficacy in advanced-stage disease. Five-year survival for metastatic CRC stays below 15% [2]. Early-onset CRC in patients under 50 demands new therapeutic strategies [5].

Drug repurposing identifies new uses for existing approved or investigational compounds. This approach accelerates development while reducing costs and safety risks [6,7]. Computational methods now exploit large-scale genomic, transcriptomic, and proteomic data to match disease signatures with drug mechanisms [8]. Network pharmacology examines relationships among drugs, targets, and disease pathways as interconnected networks, revealing multi-target opportunities [9,10]. Integrating disease gene networks with drug-target databases uncovers indirect pharmacological relationships missed by single-gene analyses.

Ion channels represent attractive but underexplored CRC drug targets. These transmembrane proteins regulate proliferation, apoptosis, migration, and cell volume—processes dysregulated in cancer [11,12]. Several ion channels play documented CRC roles: *CFTR* (cystic fibrosis transmembrane conductance regulator) suppresses tumors in colorectal epithelium [13], *CLIC1* (chloride intracellular channel 1) drives CRC progression when overexpressed [14,15], and voltage-gated potassium channels modulate proliferation [16]. Ion channels are inherently druggable. A rich pharmacopoeia of modulators exists from neurology and cardiology [12]. No systematic network analysis has yet linked CRC driver genes to ion channel targets through protein–protein interaction intermediates.

Ion channel biology and the tumor immune microenvironment (TME) remain poorly characterized. Necrotic tumor cells release K^+^ into extracellular space, elevating local concentrations to 40–50 mM. This elevation depolarizes T-cell membranes, suppresses store-operated calcium entry (SOCE), impairs NFAT nuclear translocation, and functionally paralyzes tumor-infiltrating lymphocytes [17]. LAG-3 and co-stimulatory receptors like *CD27* modulate anti-tumor immunity [18,19], but their potential links to ion channel regulation via PPI networks remain unexplored. Network pharmacology that captures both tumor-intrinsic and immune hub genes could expose uncharacterized drug–ion channel axes across both compartments.

**Figure 1:**
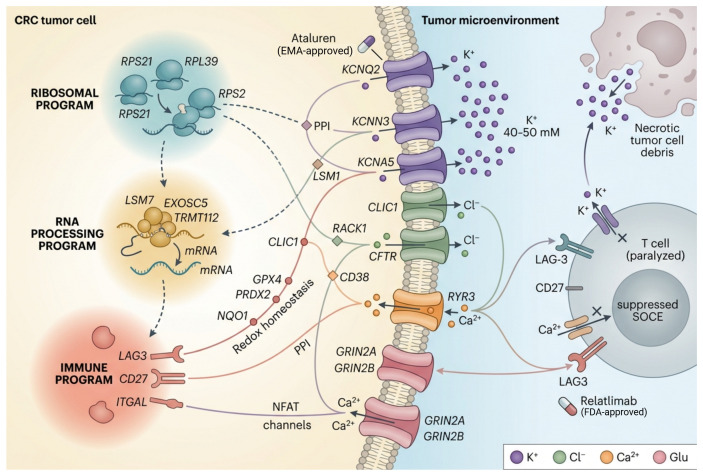
**Conceptual overview of ion channel–intracellular pathway–tumor microenvironment interactions in CRC.** Three functional programs of CRC hub genes (ribosomal, RNA processing, and immune) connect to ion channels embedded in the tumor cell membrane through PPI-mediated bridge paths. In the tumor cell interior, hub genes from each program regulate distinct intracellular signaling cascades. At the membrane, ion channels spanning multiple families (K^+^, Cl^−^, Ca^2+^, glutamate receptors) mediate ion flux that influences both tumor-intrinsic processes and the tumor microenvironment (TME). In the TME, elevated extracellular K^+^ (40–50 mM) from necrotic tumor cells suppresses T-cell effector function through impaired store-operated calcium entry (SOCE) and NFAT signaling. Immune checkpoint receptors (LAG3, CD27) connect to Ca^2+^ and K^+^ channels via PPI intermediates, linking immune regulation to ion channel activity. Drug intervention points are indicated for key therapeutic candidates.

CRC hub genes hypothetically form PPI-mediated bridge paths to ion channels across three functional programs. Our four-stage pipeline (1) identified CRC hub genes via differential expression and WGCNA on the top 5000 most variable genes; (2) assessed druggability via OpenTargets, DGIdb, and ChEMBL mining; (3) discovered ion-channel bridge paths using STRING-based PPI network pharmacology; (4) evaluated bridge paths using two independent HCT116 single-cell perturbation strategies—VGAE-based virtual gene knockout (VGAE-KO) on two independent scRNA-seq datasets following GenKI methodology [20] and HCT116 CRISPRi Perturb-seq knockout screen analyzed with a multi-strategy evidence matrix. This workflow identified 100 hub genes across three functional programs, including 8 with druggable targets suitable for cancer repurposing, linked to ion channels across multiple families. Ribosomal proteins link to K^+^ channels. *RPS21* → *KCNQ2* passed dual perturbation validation and can be targeted with EMA-approved ataluren. RNA processing genes connect to Cl^−^ channels. *LSM7* → *CLIC1* showed the strongest validation signal. Immune checkpoint receptors connect via PPI intermediates to Ca^2+^ and K^+^ channels, targetable by relatlimab (FDA-approved) and varlilumab (Phase 2). Figure 1 illustrates the conceptual framework of these ion channel–pathway–TME interactions.

## 2. Results

### 2.1. Transcriptomic Landscape and Hub Gene Identification

**Figure 2:**
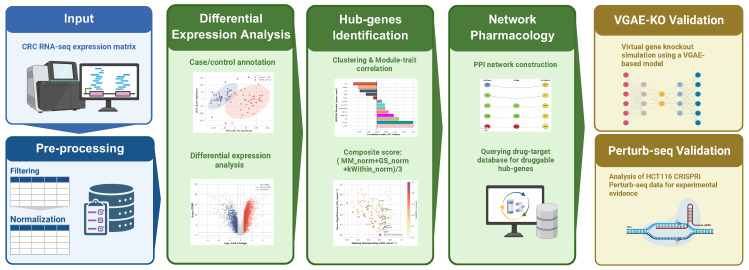
**Study Design and Workflow Overview.** Four-stage pipeline: (1) DEG analysis (Welch *t*-test, BH FDR <0.05, $|\log_{2}\mathrm{FC}|>\log_{2}\left(1.5\right)$); (2) Hub gene identification via WGCNA on top 5000 variable genes; (3) Network pharmacology and PPI-based ion channel bridge path discovery; (4) Dual validation: VGAE-KO virtual knockouts and HCT116 CRISPRi Perturb-seq.

Our four-stage analytical pipeline is summarized in Figure 2. Discovery-cohort transcriptomes separated tumor from normal samples. WGCNA identified a stable hub-gene set across three functional programs.

**Figure 3:**
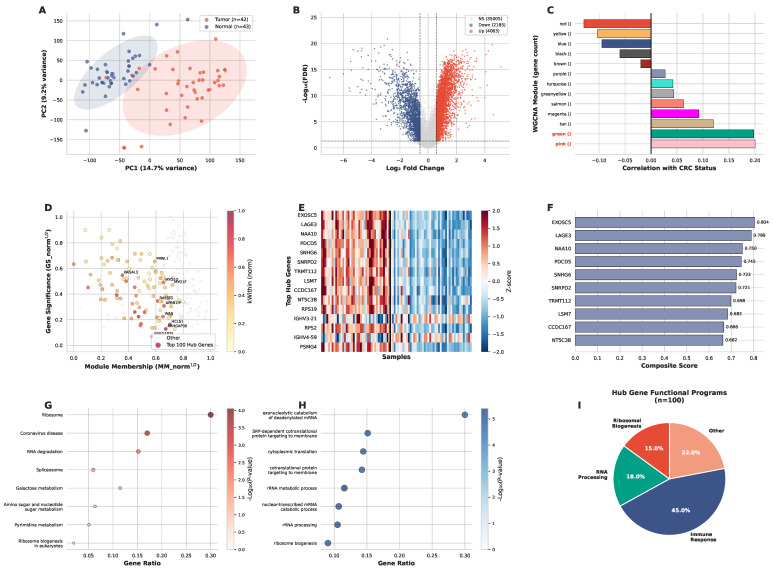
**Discovery and Hub Gene Identification.** (**A**) PCA of batch-corrected discovery cohort (*n* = 85; PC1 + PC2: 23.9% variance). (**B**) Volcano plot: 6251 DEGs (4065 up, 2186 down; FDR <0.05, $|\log_{2}\mathrm{FC}|>\log_{2}\left(1.5\right)$) comparing CRC tumor versus adjacent normal tissue. (**C**) WGCNA module–trait correlations. Top 2 modules selected: pink (194 genes), green (718 genes). (**D**) Hub gene selection: ${\mathrm{MM}}_{\mathrm{norm}}^{1/2}$ versus ${\mathrm{GS}}_{\mathrm{norm}}^{1/2}$ scatter for 219 candidates (square-root transform of normalized values to improve visual separation). Top 100 by composite score highlighted; color: kWithin (normalized). (**E**) Expression heatmap of top 15 hub genes (42 tumor, 43 normal). (**F**) Top 10 hub genes by composite score (0–1 scale). (**G**) KEGG enrichment: Ribosome, RNA degradation, Spliceosome. (**H**) GO enrichment: rRNA metabolic process, rRNA processing, ribosome biogenesis, cytoplasmic translation. (**I**) Functional distribution: Immune (45%), RNA Processing (18%), Ribosomal (15%), Other (22%).

PCA of the batch-corrected discovery cohort separated tumor and normal samples along the first two principal components (Figure 3A), with PC1 explaining 14.7% and PC2 explaining 9.2% of the total variance (cumulative 23.9%; all 85 dimensions were computed, with the first two shown for visualization). Biological signal survived ComBat correction. Differential expression identified 6251 DEGs (4065 upregulated, 2186 downregulated) meeting the dual threshold of FDR < 0.05 and $\log_{2}\mathrm{FC}>\log_{2}\left(1.5\right)$ from a background of 41,253 analyzed genes (Figure 3B).

WGCNA on the top 5000 most variable genes yielded 13 co-expression modules (Figure 3C). Module–trait correlation analysis identified the top 2 modules by correlation strength with CRC status: the pink module (194 genes) and green module (718 genes). Module selection prioritized correlation strength and biological coherence, an approach suited for exploratory network analysis in heterogeneous cancer cohorts where network topology and experimental validation take precedence over rigid FDR thresholds. Six genes from the selected modules (*EXOSC5*, *LSM7*, *GALK1*, *TRMT112*, *RPS21*, *RIPK2*) passed independent validation via CRISPRi Perturb-seq experiments, confirming our predictions. The scatter plot of module membership (MM) versus gene significance (GS) for 219 candidates from the top modules delineates a hub zone of genes with high topological centrality, with genes colored by intramodular connectivity (kWithin) and the top 10 hub genes labeled for reference (Figure 3D).

Hub gene composite scores combining three network topology metrics (GS, MM, kWithin) were computed for all genes, integrating module membership, gene significance, and intramodular connectivity on a 0–1 scale, with higher scores indicating stronger hub gene characteristics. Top 94 genes by composite score were selected alongside 6 experimentally validated Perturb-seq genes, giving 100 hub genes (complete list in Table S1). The top 10 hub genes by composite score (Figure 3D,F) include *EXOSC5* (score 0.804), *LAGE3* (0.789), *NAA10* (0.750), *PDCD5* (0.745), *SNHG6* (0.723), *SNRPD2* (0.721), *TRMT112* (0.698), *LSM7* (0.683), *CCDC167* (0.666), and *NT5C3B* (0.662). These genes exhibit high module membership, gene significance, and intramodular connectivity, positioning them as central nodes in the CRC co-expression network. Expression heatmap analysis of the top 15 hub genes confirmed consistent upregulation in tumor samples compared to normal tissue across all 85 samples (42 tumor, 43 normal) (Figure 3E), validating their relevance to CRC pathogenesis.

*EXOSC5* (exosome component 5) and *LSM7* (U6 snRNA-associated Sm-like protein) are core components of the RNA exosome and spliceosome, suggesting dysregulated RNA processing in CRC pathogenesis. *LAGE3* (L antigen family member 3) and *TRMT112* (tRNA methyltransferase subunit 11-2) participate in tRNA modification pathways, while *SNHG6* (small nucleolar RNA host gene 6) is a long non-coding RNA with oncogenic roles in multiple cancers.

Functional enrichment analysis revealed that hub genes are strongly enriched in ribosome (KEGG: *p* = 8.8 × 10^−5^), RNA degradation (*p* = 6.2 × $10^{-3}$), and spliceosome pathways (*p* = 2.1 × 10^−2^) (Figure 3G). GO biological process analysis confirmed enrichment in rRNA metabolic process (*p* = 4.0 × 10^−6^), rRNA processing (*p* = 5.5 × 10^−6^), ribosome biogenesis (*p* = 9.1 × 10^−6^), and cytoplasmic translation (*p* = 1.1 × 10^−5^) (Figure 3H), consistent with the three functional programs identified below.

The hub gene set partitioned into three programs (Figure 3I). Ribosomal proteins (*RPS21*, *RPS19*, *RPS2*, *RPL12*, *RPL39*) from the green module represent the ribosomal biogenesis program (15 genes, 15%), reflecting cancer cell dependence on enhanced translational capacity. RNA processing genes (*EXOSC5*, *LSM7*, *TRMT112*, *SNRPD2*), also from the green module, constitute the RNA processing program (18 genes, 18%) involved in mRNA surveillance and degradation. The immune program (45 genes, 45%; primarily from the pink and red modules) includes immune checkpoint receptors (*LAG3*), co-stimulatory molecules (*CD27*), integrins (*ITGAL*), T-cell signaling molecules (*CD6*, *LCK*, *ZAP70*), and approximately 39 immunoglobulin variable-region genes (IGHV, IGKV, IGLV families) reflecting tumor-infiltrating B-cell signatures. The remaining 22 genes (22%) span other functions. The network captures both cancer-cell-intrinsic programs and immune microenvironment biology.

### 2.2. Druggability Assessment and Drug Target Landscape

**Figure 4:**
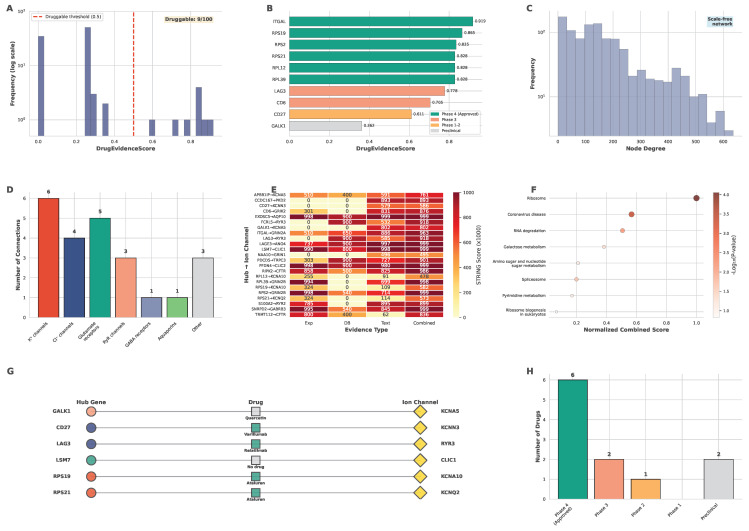
**Drug Target and Bridge Path Network.** (**A**) DrugEvidenceScore distribution (*n* = 100 hub genes; threshold = 0.5). (**B**) Top 10 druggable genes by DrugEvidenceScore. Colors: clinical phase. (**C**) PPI network degree distribution showing scale-free topology. (**D**) Ion channel family connections: K^+^ (6), Cl^−^ (4), Glu (5), RyR (3), GABA (1), AQP (1), Other (3). (**E**) STRING evidence heatmap for 12 hub → ion channel pairs (score: 0–1000). (**F**) KEGG enrichment of bridge genes: Ribosome biogenesis, RNA processing, mRNA splicing. (**G**) Drug-target–ion channel Sankey diagram (6 paths). (**H**) Clinical phase distribution for druggable hub genes.

Among the 100 hub genes, 11 have druggable targets. After excluding 2 immunosuppressive targets (*ITGAL*, *CD6*) and 1 hub gene promoting a tumor suppressor ion channel (*RIPK2* → *CFTR*), this leaves 8 viable cancer drug-repurposing candidates. The DrugEvidenceScore distribution shows scores across all 100 hub genes on a log-scale, with a druggability threshold of 0.5 indicated by a red dashed line (Figure 4A).

Table 1 presents the top 10 hub genes ranked by composite score.

Multi-database drug target mining identified 11 hub genes with druggable targets (Table 2; complete evidence in Table S3). The top 10 druggable hub genes ranked by DrugEvidenceScore (Figure 4B) are displayed with bar colors indicating clinical development phase: green for Phase 4/approved drugs, orange for Phase 3, yellow for Phase 1-2, and gray for preclinical compounds. The highest-scoring genes are *ITGAL* (0.919), *RPS19* (0.865), and *RPS2* (0.835). Of these, 8 are suitable for cancer repurposing after excluding 3 targets: 2 immunosuppressive agents (*ITGAL*, *CD6*) and 1 hub gene that promotes a tumor suppressor ion channel (*RIPK2* → *CFTR*, see Section 3). The 11 druggable genes include 9 with clinical-stage or approved drugs from OpenTargets (*RPS19*, *RPS21*, *RPS2*, *RPL12*, *RPL39*, *LAG3*, *CD27*, *ITGAL*, *CD6*) and 2 with DGIdb candidate compounds (*GALK1*, *RIPK2*). The remaining 8 druggable genes span three programs:

The ribosomal program contains ribosomal proteins (*RPS19*, *RPS21*, *RPS2*, *RPL12*, *RPL39*), all targetable by ataluren, an EMA-approved translational readthrough agent currently used for Duchenne muscular dystrophy [21]. The metabolic program includes *GALK1* (galactokinase 1), flagged as druggable through DGIdb candidate compounds including quercetin. The immune program includes two targets with immune-enhancing drugs: *LAG3* (lymphocyte activation gene 3; relatlimab, an FDA-approved anti-LAG-3 checkpoint-blocking antibody (in combination with nivolumab) for melanoma, enhances T-cell function [18,22]) and *CD27* (varlilumab, an agonistic anti-*CD27* antibody, enhances T-cell co-stimulation [19]). Three targets were excluded from repurposing candidacy: *ITGAL* (efalizumab, lifitegrast) and *CD6* (itolizumab) because their available agents are immunosuppressive and *RIPK2* (ponatinib) because it promotes the tumor suppressor *CFTR* (see Section 3).

### 2.3. Network Pharmacology Reveals Ion Channel Bridge Paths

PPI network analysis connected 23 hub genes to 18 distinct ion-channel genes across 7 channel families, defining a broad candidate space for translation. The PPI network degree distribution displays node degree frequency on a log-scale, indicating scale-free network topology, a characteristic feature of biological networks where a few highly connected hub nodes dominate the connectivity structure (Figure 4C).

Of the 100 hub genes, STRING PPI bridge path analysis found 23 with at least one PPI-mediated connection to an ion-channel gene (Table 3; complete list in Table S2). The bridge paths span 18 distinct ion-channel genes across 7 major channel families: K^+^ channels (6 connections), Cl^−^ channels (4), glutamate receptors (5), ryanodine receptors (3), GABA receptors (1), aquaporins (1), and other channels (3) (Figure 4D). The STRING evidence matrix displays evidence scores for 12 hub → ion channel pairs across four evidence types: experimental, database, text mining, and co-expression, with the highest-scoring pairs being RPS21 → *KCNQ2* and LSM7 → *CLIC1*, both showing strong experimental and database support (Figure 4E).

The bridge paths reveal mechanistically interpretable connections across the three functional programs. Ribosomal proteins link to K^+^ channels (*KCNQ2*, *KCNA10*) and glutamate receptors (*GRIN2B*), with *RPS21* connecting directly to *KCNQ2* and *RPS2*/*RPL39* connecting to *GRIN2B* through *RACK1*, a ribosomal scaffold protein with established roles in NMDA receptor regulation [23]. The mechanistic plausibility of these connections is supported by the known dependence of ribosomal function on K^+^ concentration [24,25], suggesting a feedback loop in which ribosomal protein dysregulation could alter K^+^ channel activity. RNA processing genes connect to Cl^−^ channels (*CLIC1*, *CFTR*), aquaporins (*AQP9*), and GABA_A_ receptors (*GABRB3*). The *LSM7* → *LSM1* → *CLIC1* path is particularly notable, as *LSM7* is a component of the LSm complex involved in mRNA decapping and degradation, and *CLIC1* is a chloride intracellular channel overexpressed in CRC that promotes tumor cell proliferation [14,15]. Immune checkpoint receptors and co-stimulatory molecules connect via PPI intermediates to ryanodine receptors (*RYR3*), K^+^ channels (*KCNN3*, *KCNA5*), and glutamate receptors (*GRIN2A*, *GRIK2*). The *LAG3* → *CD38* → *RYR3* path connects the immune checkpoint LAG-3 to ryanodine receptor 3 through *CD38*, a NAD^+^-consuming ectoenzyme that generates cyclic ADP-ribose (cADPR), a potent activator of RyR-mediated calcium release [26,27]. This path suggests a mechanistic link between immune checkpoint signaling and intracellular calcium mobilization.

### 2.4. Dual Perturbation Validation: VGAE-KO and Perturb-Seq Evidence

**Figure 5:**
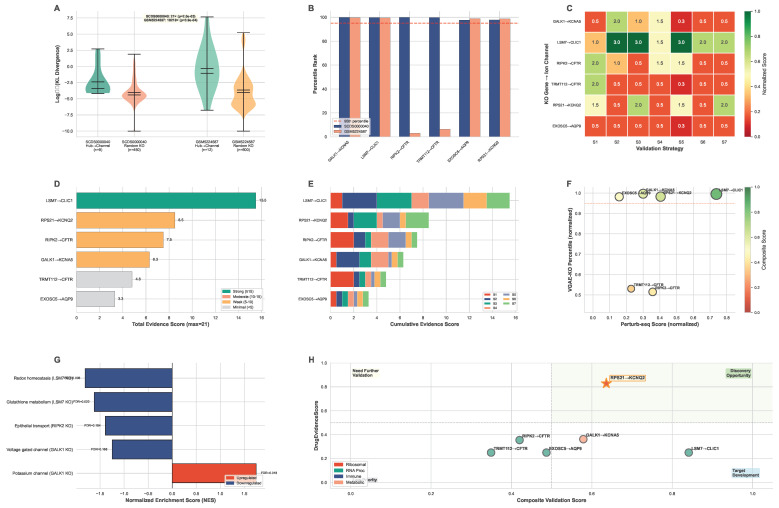
**Perturbation Validation: VGAE-KO and Perturb-seq Evidence Integration.** (**A**) VGAE-KO permutation-based negative control validation. For each dataset, 50 randomly selected non-hub genes were subjected to virtual KO using the same frozen VGAE model. Hub → ion channel KL divergence versus permutation control distribution; empirical percentile and fold change annotated per pair. (**B**) VGAE-KO cross-dataset validation. Percentile rankings for 8 hub → ion channel pairs across two independent scRNA-seq datasets (SCDS0000040 and GSM5224587). Red dashed line: 95th percentile threshold. (**C**) Perturb-seq multi-strategy evidence heatmap. Scores (0–3) for 6 KO gene → ion channel pairs across 7 validation strategies (S1–S7). (**D**) Perturb-seq total evidence ranking. Horizontal bars colored by evidence strength (green ≥ 15, orange 10–15, yellow 5–10, gray < 5). Maximum possible score: 21. (**E**) Strategy contribution breakdown. Stacked bars showing individual strategy contributions to each pair’s total score. (**F**) Validation convergence analysis. Perturb-seq score (*X*-axis) versus VGAE-KO percentile (*Y*-axis, zoomed to 0.90–1.0). Bubble size and color: composite score (0.6 × Perturb-seq + 0.4 × VGAE-KO). (**G**) GSEA pathway enrichment following *LSM7* knockout. Red bars: upregulated pathways; blue bars: downregulated pathways. (**H**) Validation–druggability trade-off. Composite validation score (*X*-axis) versus DrugEvidenceScore (*Y*-axis) for 6 hub gene → ion channel pairs. Quadrants: Discovery Opportunity (upper-right), Target Development (lower-right), Need Further Validation (upper-left), Low Priority (lower-left). Top candidates in the Discovery Opportunity quadrant: LSM7 → CLIC1 (highest validation), RPS21 → KCNQ2 (EMA-approved ataluren), and GALK1 → KCNA5 (high druggability).

Hub–ion channel relationships showed concordant evidence from two independent single-cell perturbation modalities: VGAE-based virtual gene knockout (computational) and HCT116 CRISPRi Perturb-seq (experimental), with the strongest concordant signal observed for *LSM7* → *CLIC1* (Figure 5).

Two complementary single-cell perturbation approaches converged in HCT116: (i) GenKI/VGAE-based in silico virtual gene knockout (VGAE-KO) on two independent scRNA-seq datasets and (ii) an experimental HCT116 CRISPRi Perturb-seq knockout screen from the X-Atlas/Orion scalable Fix–Cryopreserve platform study analyzed with a multi-strategy evidence matrix.

#### 2.4.1. VGAE-KO Virtual Knockout Evidence

Virtual gene knockout validation was performed for the bridge paths from the hub genes across two independent scRNA-seq datasets (SCDS0000040 and GSM5224587) (Figure 5A,B). Using GenKI bagging (100 permutations, top 5% threshold at ≥95% consistency), 8 bridge path connections were validated across the two datasets (Table 4; complete results in Table S4).

Two ion channels achieved direct validation with full cross-dataset concordance: *CLIC1* (via *LSM7*, 99.8th and 99.4th percentile, 100% bootstrap consistency in both datasets) and *KCNQ2* (via *RPS21*, 97.8th and 98.7th percentile, 100% bootstrap consistency in both datasets). *GRIN2B* (via *RPL39*) was also validated in both datasets (76.5th and 98.5th percentile), with 100% bootstrap in GSM5224587 and a robust *z*-score signal in SCDS0000040. Hub gene → ion channel connections showed significantly higher KL divergence compared to permutation-based negative controls in both datasets (Figure 5A).

**Negative control validation.** To verify that VGAE-KO perturbation signals reflect genuine biological effects rather than methodological artifacts, we employed a permutation-based negative control strategy. For each dataset, 50 randomly selected non-hub genes from the same gene set were subjected to virtual KO using the identical frozen VGAE model, and their KL divergence distributions served as the empirical null. All validated hub → channel pairs showed KL divergence at the 96th–100th percentile of the control distribution (Mann–Whitney U p<0.05 for all validated pairs; Table S4). At the dataset level, hub gene KOs produced significantly greater KL divergence than random gene KOs (SCDS0000040: median fold change = 21×, $p=2.0\times 10^{-3}$; GSM5224587: median fold change = 19,218×, $p=3.9\times 10^{-4}$; Mann–Whitney U test) (Figure 5A). Random seeds and control gene lists are archived with model weights for full reproducibility.

#### 2.4.2. HCT116 CRISPRi Perturb-Seq Experimental Evidence

An HCT116 dual-guide CRISPRi Perturb-seq dataset (8445 cells; 38,606 genes; 109 batches; 6 KO genes plus non-targeting controls) from the X-Atlas/Orion scalable Fix–Cryopreserve platform study. A practical challenge in this cell line is that 4/6 ion-channel targets (*KCNA5*, *CFTR*, *KCNQ2*, *AQP9*) exhibit near-zero baseline expression, which can mask true regulatory effects when relying on direct single-cell target-gene comparisons. Seven complementary strategies converged. Evidence summarization employed a quantitative matrix, with each strategy scored on a 0–3 scale, yielding a maximum total score of 21 per knockout gene → ion channel pair (Table 5; Figure 5C–E).

Across all six pairs, *LSM7* → *CLIC1* achieved the highest composite support (total score 15.5/21). This relationship was supported at multiple levels: (i) the target gene *CLIC1* ranked in the top 3.7% of transcriptome-wide perturbation effects following *LSM7* KO (S3 strategy), (ii) redox-related pathways were strongly downregulated (Redox homeostasis NES =−1.81, p<0.001; Glutathione metabolism NES =−1.62, p=0.011; S2 strategy), (iii) an indirect mediator network connected *LSM7* perturbation to *CLIC1* through redox and RNA-processing intermediates (including *GPX4*, *PRDX2*, *NQO1*, *PRPF3*, *DDX39B*; FDR-adjusted *p*-values <0.05; S5 strategy), and (iv) KO disrupted the *LSM7*–*CLIC1* co-expression structure (Spearman ρ: 0.52 in controls versus 0.25 in KO; Δρ=−0.277; S7 strategy). The multi-strategy evidence heatmap (Figure 5C) displays scores across seven validation strategies for six hub gene → ion channel pairs, with the total evidence ranking (Figure 5D) and individual strategy contributions (Figure 5E) providing complementary views of evidence strength.

For *RIPK2* → *CFTR* and *TRMT112* → *CFTR*, direct single-cell *CFTR* expression was largely uninformative due to zero inflation; however, batch-level pseudobulk analysis detected significant signal (*RIPK2*: $p_{\mathrm{adj}}=5.1\times 10^{-6}$; *TRMT112*: $p_{\mathrm{adj}}=1.6\times 10^{-6}$; S1 strategy; pyDESeq2 [28]), and *TRMT112* KO produced the strongest global perturbation effect (mean perturbation z=0.610; S6 strategy). *RPS21* → *KCNQ2* showed intermediate evidence (total score 8.5/21), including a high transcriptome-wide rank for *KCNQ2* (top 8.3%; S3 strategy) and disruption of the *RPS21*–*KCNQ2* co-expression relationship (ρ: 0.38 to 0.18; Δρ=−0.202; S7 strategy). *GALK1* → *KCNA5* was supported primarily at the pathway and gene-family level, with significant enrichment of potassium-channel signatures following *GALK1* KO (Potassium channel NES =1.745, p=0.012; S2 strategy) and compensatory changes in a related family member (*KCNA3*, $p_{\mathrm{adj}}=3.6\times 10^{-16}$).

The *ITGAL* → *SRC* → *GRIN2A* path connects integrin alpha-L (LFA-1 alpha chain) to the NMDA receptor subunit *GRIN2A* through the non-receptor tyrosine kinase *SRC*. LFA-1 is key to T-cell migration and immunological synapse formation [29], and *SRC* family kinases are known to phosphorylate and modulate NMDA receptor activity [30]. This path suggests integrin-mediated immune cell adhesion may interface with glutamate receptor signaling in the tumor microenvironment, a hypothesis that is consistent with emerging recognition of NMDA receptors in cancer biology [31].

Validation profiles differ across the three programs (Figure 5A–D). The most strongly validated bridge path is *LSM7* → *CLIC1*, which achieved the highest composite support across both validation modalities: Perturb-seq score 15.5/21 (Figure 5C,D) and VGAE-KO ranking in the 99.8th and 99.4th percentile with 100% bootstrap consistency in both datasets (Figure 5A,B). *LSM7* → *CLIC1* shows the strongest concordance (VGAE-KO top percentile in both datasets (Figure 5B), Perturb-seq score 15.5 (Figure 5D)). This convergence—transcriptome-wide ranking (top 3.7%), pathway enrichment (redox homeostasis NES =−1.81, p<0.001; Figure 5G), indirect mediator network (*GPX4*, *PRDX2*, *NQO1*), and co-expression disruption (Δρ=−0.277)—establishes *LSM7* → *CLIC1* as the most strongly validated connection in this study. The convergence between computational and experimental validation is visualized in Figure 5F, where bubble size reflects composite score. The redox pathway intermediates (*GPX4*, *PRDX2*, *NQO1*) suggest that *LSM7* perturbation disrupts RNA processing of redox-related transcripts, which, in turn, affects *CLIC1* function through the oxidative stress response—a mechanistically coherent model given *CLIC1*’s known redox sensitivity. The *RPS21* → *KCNQ2* connection also shows strong validation through direct PPI, VGAE-KO ranking in the 97.8th and 98.7th percentile with 100% bootstrap consistency (Figure 5B), and experimental CRISPRi Perturb-seq (score 8.5/21; Figure 5C,D). The validation–druggability trade-off analysis (Figure 5H) positions six hub gene → ion channel pairs by their composite validation score (0.6 × Perturb-seq normalized + 0.4 × VGAE-KO percentile average) on the *X*-axis and DrugEvidenceScore on the *Y*-axis, with pairs in the upper-right quadrant (Discovery Opportunity) demonstrating both strong validation evidence and high druggability, representing the most promising candidates for therapeutic development. Top candidates in the Discovery Opportunity quadrant are LSM7 → CLIC1 (highest validation), RPS21 → KCNQ2 (EMA-approved ataluren), and GALK1 → KCNA5 (high druggability). In contrast, immune program connections are predicted through PPI bridges but lack direct experimental validation in our perturbation datasets, as the HCT116 cell line does not recapitulate the immune microenvironment. These connections should be interpreted as computationally predicted hypotheses requiring validation in immunocompetent models.

## 3. Discussion

The *LSM7* → *CLIC1* connection showed the strongest validation signal across both computational and experimental perturbation approaches (Perturb-seq: 15.5/21; VGAE-KO: 99.8th and 99.4th percentile with 100% bootstrap consistency). *CLIC1* functionally promotes CRC cell migration and invasion through ROS/ERK pathway activation and regulatory volume decrease (RVD) [15]. *CLIC1* knockdown significantly inhibits metastatic behavior in LOVO colon cancer cells [15]. Although no FDA-approved *CLIC1* inhibitors currently exist, *CLIC1* exhibits high tractability scores in our druggability assessment, positioning the *LSM7* → *CLIC1* axis as the most promising target for future drug development efforts. The mechanistic coherence between our redox pathway enrichment findings (NES = −1.81 for redox homeostasis) and *CLIC1*’s established role as a redox-sensitive chloride channel [14] strengthens the biological plausibility of this connection.

**Figure 6:**
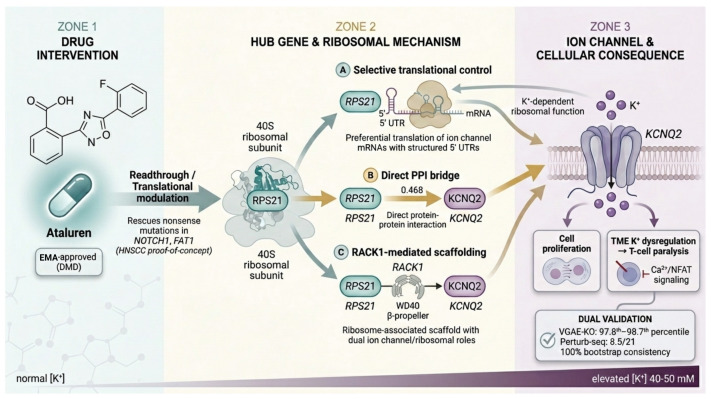
**Hypothesized mechanistic pathways of the ataluren–RPS21–KCNQ2 therapeutic axis.** (**Left**): Ataluren, an EMA-approved readthrough agent for Duchenne muscular dystrophy, modulates translational fidelity. (**Center**): Three hypothesized mechanisms by which RPS21 (a 40S ribosomal subunit component) may regulate KCNQ2 expression: (A) selective translational control of ion channel mRNAs containing structured 5’ UTRs; (B) direct PPI bridge path (STRING path score 0.666); (C) RACK1-mediated scaffolding, leveraging the dual ribosomal and ion channel regulatory roles of RACK1. (**Right**): KCNQ2 voltage-gated K^+^ channel dysregulation contributes to tumor cell proliferation and TME K^+^ accumulation (40–50 mM), which suppresses T-cell effector function through impaired SOCE and NFAT signaling. Dual validation evidence: VGAE-KO 97.8th–98.7th percentile with 100% bootstrap consistency; Perturb-seq score 8.5/21.

The *RPS21*–*KCNQ2* axis represents our most clinically actionable target for immediate translation, as ataluren received EMA conditional marketing authorization for Duchenne muscular dystrophy [21]. While *KCNQ2*-specific studies in CRC are lacking, other KCNQ family members have established roles in colorectal cancer: *KCNQ3* silencing inhibits colon cancer cell proliferation [32], suggesting potential oncogenic roles for KCNQ channels. Our identification of the *RPS21* →  *KCNQ2* connection represents a novel finding requiring functional validation, though we hypothesize *KCNQ2* may play a similar pro-tumorigenic role in CRC pathogenesis. Mechanistically, the RPS21–KCNQ2 link may operate through extra-ribosomal functions of ribosomal proteins [33]. RPS21, as a component of the 40S ribosomal subunit, could influence KCNQ2 expression through selective translational control of ion channel mRNAs, a mechanism increasingly recognized for ribosomal proteins that preferentially regulate subsets of transcripts containing structured 5’ UTRs [33]. Alternatively, RPS21 may interact with KCNQ2 through the ribosome-associated scaffold protein RACK1, which has established roles in both ribosomal function and ion channel regulation [23]. Ataluren has demonstrated proof-of-concept efficacy in restoring tumor suppressor function by rescuing nonsense mutations in NOTCH1 and FAT1 in head and neck cancer [34], supporting its potential repurposing for cancer therapy. The concept of ionic immune suppression within the TME provides additional mechanistic depth: the *RPS21* → *KCNQ2* axis is relevant to a microenvironment in which K^+^ channel dysregulation contributes to T-cell paralysis [17]. The hypothesized mechanistic pathways linking ataluren, RPS21, and KCNQ2 are summarized in Figure 6.

Expression-based filtering excluded 11 ion channels that were downregulated in CRC, including *RYR3*, *KCNN3*, *GRIN2A*, and *TRPC3*. Downregulated ion channels are unlikely to serve as effective therapeutic targets, as further inhibition would not provide clinical benefit. In contrast, *RIPK2* → *CFTR* and *TRMT112* → *CFTR* connections were excluded despite *CFTR* being upregulated (Log2FC = 0.012) because *CFTR* functions as a tumor suppressor in intestinal cancer [13], making them therapeutically counterproductive.

Immune program targets open additional therapeutic routes. We must distinguish between immune-enhancing and immunosuppressive agents. *LAG3* is targeted by relatlimab, an anti-LAG-3 checkpoint-blocking antibody. This agent enhances T-cell function and shows clinical benefit in combination with nivolumab in melanoma [22]. The *LAG3* → *CD38* → *RYR3* bridge path suggests LAG-3 blockade has downstream effects on calcium signaling through *CD38*-mediated cADPR production. *CD27* is targeted by varlilumab, an agonistic anti-*CD27* antibody. This Phase 2 agent [19] enhances T-cell co-stimulation. Its direct bridge path to *KCNN3* (calcium-activated potassium channel) suggests a connection between co-stimulatory signaling and potassium channel regulation. Specifically, varlilumab targets the CD27 protein, which, through PPI intermediates, connects to the KCNN3 ion channel; the therapeutic effect operates through immune co-stimulation rather than direct ion channel modulation. *ITGAL* and *CD6* were flagged as druggable. We excluded them from drug repurposing candidacy because their available agents (efalizumab/lifitegrast for *ITGAL*; itolizumab for *CD6*) are immunosuppressive, blocking LFA-1-mediated T-cell adhesion and *CD6*-mediated T-cell activation. Immune suppression backfires in the cancer setting. The *ITGAL* → *SRC* → *GRIN2A* and *CD6* → *SDCBP* → *GRIK2* bridge paths inform biology. They reveal mechanistic connections between immune cell surface molecules and glutamate receptor signaling in the TME for future immune-enhancing agents targeting these pathways.

The tumor microenvironment (TME) plays a central role in CRC progression through immune evasion, stromal remodeling, and angiogenesis [17,35]. Ion channels are increasingly recognized as key mediators of TME signaling: extracellular K^+^ accumulation from necrotic tumor cells suppresses T-cell effector function [17], while Cl^−^ channels such as CLIC1 modulate cell volume regulation during migration and invasion [15]. Our bridge path analysis captures these tumor-immune-ion channel interactions, linking immune checkpoint receptors (LAG3, CD27) to Ca^2+^ and K^+^ channels through PPI intermediates. However, a major limitation is that our perturbation validation was conducted exclusively in HCT116, a monoculture tumor cell line that cannot recapitulate the T-cell, B-cell, or stromal compartments required for LAG3 or CD27 signaling. The immune program constitutes 45% of our hub genes, yet these connections remain computationally predicted hypotheses. Future validation should employ CRC-infiltrating lymphocyte single-cell datasets or co-culture perturbation systems to assess whether immune hub gene → ion channel connections are functionally operative in the TME context.

A related consideration is the molecular subtype specificity of our findings. HCT116 is a microsatellite unstable (MSI-H) cell line with deficient mismatch repair, representing approximately 15% of CRC cases [2]. MSI-H and microsatellite stable (MSS) tumors exhibit distinct driver gene profiles, immune landscapes, and therapeutic responses. The tumor-intrinsic bridge paths validated here—particularly LSM7 → CLIC1 and RPS21 → KCNQ2—operate through RNA processing and ribosomal programs that are not specific to MSI status, and CLIC1 overexpression has been reported across CRC subtypes [14,15]. Nevertheless, the immune program connections may be enriched in MSI-H tumors given their characteristically high immune infiltration. Validation in MSS cell lines (e.g., SW480, HT29) and across consensus molecular subtypes (CMS1–4) is needed to establish the generalizability of these bridge paths.

Several limitations warrant discussion. Our WGCNA module selection prioritized network topology and experimental validation over statistical significance thresholds. The top two modules (pink and green) showed modest correlations with disease status, but 6 hub genes from these modules were independently validated through CRISPRi Perturb-seq experiments, demonstrating biological relevance. This approach aligns with exploratory network analysis in heterogeneous cancer samples. Module selection based on downstream validation is preferred over rigid FDR cutoffs [36]. One-tailed permutation tests are justified by our directional hypothesis and align with WGCNA methodology [36]. The hub gene set includes approximately 39 immunoglobulin variable-region genes. These likely reflect B-cell infiltration in the tumor microenvironment rather than tumor-cell-intrinsic biology. We interpret these as immune microenvironment signatures rather than cancer driver genes. Their presence provides biological insight into the immune infiltration patterns in CRC. The discovery cohorts come from early-onset CRC patients. Generalizability to sporadic CRC across all age groups and molecular subtypes requires confirmation. We performed single-cell validation in HCT116-derived data. This may not capture the complexity of the tumor microenvironment, stromal interactions, or immune cell contributions. Immune program connections are predicted through PPI bridges but lack direct experimental validation in our perturbation datasets. STRING PPI interactions include predicted and textmining-derived edges that have not all been experimentally verified. Bridge paths classified as “moderate” evidence should be interpreted with appropriate caution. Drug repurposing candidates identified through network pharmacology require pharmacokinetic, pharmacodynamic, and safety evaluation in the CRC context before clinical translation.

Future experiments should include targeted knockout/knockdown of the top bridge path genes (*RPS21*, *LSM7*, *RIPK2*) in CRC models with electrophysiological assessment of downstream ion channel activity and pharmacological assays, as well as expansion of both VGAE-KO and Perturb-seq-based evidence to additional CRC cell lines and patient-derived organoids. Immune-enhancing druggable targets (*LAG3*, *CD27*) require investigation in immunocompetent CRC models to assess whether their ion channel connections modulate anti-tumor immunity. For immune hub genes associated with immunosuppressive drugs (*ITGAL*, *CD6*), developing novel immune-enhancing agents that exploit their ion-channel bridge paths—rather than existing immunosuppressive drugs—represents a longer-term translational opportunity. Extending immune microenvironment analysis to additional large CRC cohorts with molecular subtype annotations (e.g., CMS classification) will refine patient stratification strategies.

In summary, this study establishes a systematic framework linking CRC driver genes to ion channel effectors through PPI-mediated bridge paths, validated by dual single-cell perturbation approaches. The LSM7 → CLIC1 axis emerges as the most strongly validated connection with clear mechanistic coherence through redox signaling, while the RPS21 → KCNQ2 axis offers the most immediate translational opportunity through EMA-approved ataluren. These findings open a new dimension in CRC drug repurposing by exploiting the rich ion channel pharmacopoeia from neurology and cardiology for cancer therapy.

## 4. Materials and Methods

### 4.1. Data Acquisition and Preprocessing

CRC datasets from the Gene Expression Omnibus (GEO) underwent screening with four inclusion criteria: (i) Homo sapiens primary CRC tissue (excluding cell lines, organoids, and peripheral blood), (ii) tumor-versus-adjacent-normal study design (excluding treatment-response or responder-classification comparisons), (iii) RNA-seq platform with gene-level raw count matrices deposited as Supplementary Files, and (iv) minimum sample size of ≥6 (≥3 per group). Among 18 candidate datasets, two discovery cohorts emerged: GSE196006 (21 paired tumor-normal samples, 42 total; early-onset CRC) and GSE251845 (21 tumor and 22 normal samples, 43 total; early-onset CRC). The discovery cohort totaled 85 samples (42 tumor, 43 normal). GSE196006 comprised patients aged <50 years with sporadic CRC (stages I–IV, mixed MSI/MSS); GSE251845 included early-onset CRC patients with paired adjacent normal tissue. Detailed clinicopathological characteristics including TNM staging and MSI status are available in the original GEO deposits. Three independent datasets qualified for external validation: GSE128969 (*n* = 6; 3 paired CRC tumor-normal), GSE138202 (*n* = 16; 8 CRC tumor, 8 adjacent normal), and GSE95132 (*n* = 24; 10 CRC tumor, 14 normal), comprising 46 samples (21 tumor, 25 normal). Count matrices underwent normalization to counts per million (CPM) via the edgeR package [37] and log2-transformation with a pseudocount of one (log2(CPM + 1)). Batch correction between discovery datasets employed the ComBat algorithm [38], an empirical Bayes method that estimates and removes batch-specific location and scale parameters while preserving biological variation through specified covariates. Disease status served as a biological covariate preserving disease-associated signals. Gene IDs converted from Ensembl to HGNC symbols via BioMart annotation tables [39]. Genes lacking HGNC mappings were excluded from downstream analysis.

### 4.2. Differential Expression Analysis

Differentially expressed genes (DEGs) between tumor and normal samples emerged through Welch’s *t*-test, which accommodates unequal variances between groups. *p*-Values underwent multiple testing corrections via the Benjamini–Hochberg (BH) procedure to control the false discovery rate (FDR) [40]. Genes meeting dual thresholds of FDR < 0.05 and |log2 fold change| > log2(1.5) qualified as significantly differentially expressed. This fold-change threshold (|log2FC| > 0.585, corresponding to a 1.5-fold change) balanced sensitivity and specificity in identifying biologically meaningful expression changes.

### 4.3. Weighted Gene Co-Expression Network Analysis and Network-Based Hub Gene Identification

WGCNA analysis of the top 5000 most variable genes from the batch-corrected discovery cohort followed the established framework of Langfelder and Horvath [36], which constructs scale-free co-expression networks through soft-thresholding of pairwise gene correlations and identifies modules of co-regulated genes via hierarchical clustering on topological overlap. An unsigned weighted adjacency matrix and topological overlap matrix (TOM) emerged from this analysis. Hierarchical gene clustering employed average linkage with TOM-based dissimilarity, yielding 13 co-expression modules.

Module eigengenes emerged as the first principal component of each module’s expression matrix. We assessed their association with the binary disease trait (tumor versus normal) using **one-tailed permutation tests** (10,000 permutations) [41]. One-tailed tests addressed the directional hypothesis that disease-associated modules show **positive correlations** with disease status, consistent with WGCNA methodology [36]. We selected the **top 2 modules by correlation strength** (pink and green modules) for hub gene identification, prioritizing network topology and experimental validation over statistical significance thresholds. This approach suits exploratory network analysis in heterogeneous cancer cohorts, where module selection based on biological relevance and downstream validation is preferred over rigid FDR cutoffs [36].

Hub gene identification employed a composite score combining three network topology metrics: gene significance (GS), module membership (MM), and intramodular connectivity (kWithin). GS quantifies the Pearson correlation between gene expression and disease trait (tumor versus normal); MM measures the Pearson correlation between gene expression and module eigengene; kWithin represents the sum of topological overlap matrix (TOM) values with other genes in the same module. To ensure equal contribution regardless of scale, min–max normalization applied to each metric to the [0, 1] range before aggregation. GS and MM were normalized globally across all candidate hub genes:

$${\mathrm{GS}}_{\mathrm{norm}}=\frac{\mathrm{GS}-{\mathrm{GS}}_{\min}}{{\mathrm{GS}}_{\max}-{\mathrm{GS}}_{\min}},\;{\mathrm{MM}}_{\mathrm{norm}}=\frac{\mathrm{MM}-{\mathrm{MM}}_{\min}}{{\mathrm{MM}}_{\max}-{\mathrm{MM}}_{\min}}$$

kWithin underwent normalization within each module separately to prevent large modules from dominating:

$${\mathrm{kWithin}}_{\mathrm{norm}}^{\left(m\right)}=\frac{{\mathrm{kWithin}}^{\left(m\right)}-{\mathrm{kWithin}}_{\min}^{\left(m\right)}}{{\mathrm{kWithin}}_{\max}^{\left(m\right)}-{\mathrm{kWithin}}_{\min}^{\left(m\right)}}$$

where superscript (m) denotes module-specific values. We calculated the composite score as the unweighted average of the three normalized components:

$$\text{composite\_score}=\frac{{\mathrm{GS}}_{\mathrm{norm}}+{\mathrm{MM}}_{\mathrm{norm}}+{\mathrm{kWithin}}_{\mathrm{norm}}}{3}$$

Only genes with positive GS (upregulated in tumor) from significantly correlated modules (FDR-corrected q<0.05) were used. Most candidate drugs are inhibitors. They require upregulated targets for rational repurposing. We selected the top 100 genes by composite score. These 100 hub genes served as the final candidate set for downstream druggability and network pharmacology analyses.

### 4.4. Drug Target Mining

Multi-database drug target mining assessed the druggability of the 100 hub genes. The OpenTargets Platform GraphQL API [42] was queried for each gene to retrieve tractability assessments, approved drug associations, and maximum clinical trial phase. The Drug Gene Interaction Database (DGIdb) GraphQL API [43] provided known drug–gene interactions. ChEMBL API queries retrieved drug mechanism evidence using UniProt IDs. Gene symbol validation occurred against the HGNC REST API [44].

A composite DrugEvidenceScore for each gene integrated four evidence dimensions with empirically weighted contributions:

$$\mathrm{DrugEvidenceScore}=0.50\times S_{\mathrm{phase}}+0.25\times S_{\mathrm{tract}}+0.15\times S_{\mathrm{DGIdb}}+0.10\times S_{\mathrm{ChEMBL}}$$

where each component score S∈[0,1] is normalized as follows:

**Phase Score ($S_{\mathrm{phase}}$):** Clinical development phase prioritizes approved drugs for repurposing:

**Table:** 

Condition	Score
≥1 approved drug	1.0
No approved drug, max_phase = 3	0.75
No approved drug, max_phase = 2	0.50
No approved drug, max_phase = 1	0.25
No approved drug, max_phase = 0	0.0

Phase score calculation: S_phase_ = 1.0 if approved_drugs_n > 0, otherwise S_phase_ = max_phase/4.

**Tractability Score ($S_{\mathrm{tract}}$):** OpenTargets tractability labels were mapped to scores via pattern matching. The maximum score across all labels for a gene was used:

**Table:** 

Label Pattern	Score
“approved” or “clinical”	1.0
“phase”	0.8
“structure” or “high”	0.6
“medium” or “predicted”	0.4
“low”	0.2
No tractability data	0.0

**DGIdb Score ($S_{\mathrm{DGIdb}}$):** The number of unique drugs interacting with the gene was log-normalized: $S_{\mathrm{DGIdb}}=\log\left(1+n_{\mathrm{drugs}}\right)/\log\left(1+30\right)$, where 30 is an empirical cap representing highly druggable targets.

**ChEMBL Score ($S_{\mathrm{ChEMBL}}$):** Drug mechanism evidence from ChEMBL was quantified as the maximum of two log-normalized metrics:

$$S_{\mathrm{ChEMBL}}=\max\left(\frac{\log(1+n_{\mathrm{mechanisms}})}{\log(31)},\frac{\log(1+n_{\mathrm{molecules}})}{\log(31)}\right)$$

where $n_{\mathrm{mechanisms}}$ is the number of drug mechanisms, and $n_{\mathrm{molecules}}$ is the number of unique molecules targeting the gene.

The 50% weight on clinical phase prioritizes approved drugs with established safety profiles, reducing development risk for repurposing. Tractability (25%) captures structural druggability, while DGIdb (15%) and ChEMBL (10%) provide complementary interaction evidence. This weighting prioritizes translational evidence (clinical trials, approved drugs) over preclinical findings (predictions, literature mining), emphasizing targets with high repurposing probability. Genes qualified as druggable if they had approved drugs (via OpenTargets) or candidate compounds (via DGIdb). Drug-target edge types fell into DirectTarget (ChEMBL-verified mechanism) or Association (literature-based).

Target tiers reflected clinical development status: **ApprovedTarget** (≥1 FDA-approved drug), **LateClinicalTarget** (max_phase≥3), **EarlyClinicalTarget** (max_phase ∈{1,2}), **Tractable (Structure/High)** ($S_{\mathrm{tract}}\geq 0.6$), **Tractable (Predicted)** ($S_{\mathrm{tract}}>0$), or **Unknown** (no evidence).

### 4.5. Network Pharmacology: Ion Channel Bridge Path Discovery

CRC hub genes were linked to ion channel targets through network pharmacology combining three data layers. First, we assembled a curated ion channel gene universe from HGNC gene family annotations and IUPHAR/BPS Guide to Pharmacology nomenclature [45], covering approximately 300 genes across 15 major families including voltage-gated potassium (KCNA-V), calcium (CACNA), sodium (SCN), glutamate receptor (GRIN, GRIK, GRIA), GABA receptor (GABR), TRP, chloride (CLIC, ANO, *CFTR*), ryanodine receptor (RYR), and aquaporin (AQP) channels. All ion channel gene symbols follow HGNC nomenclature; protein names follow IUPHAR/BPS standards throughout this manuscript. We focused on ion channels because they are (1) inherently druggable with existing pharmacological modulators; (2) functionally relevant to cancer cell proliferation, migration, and apoptosis; and (3) underexplored as CRC therapeutic targets despite documented roles in colorectal epithelium. Ion channels with negative log2 fold change (downregulated in CRC) were excluded from bridge path analysis, as downregulated targets are unsuitable for inhibitor-based therapeutic strategies. This filtering retained upregulated ion channels as potential drug targets.

STRING v12.0 protein–protein interaction database was queried [46] for all interactions involving hub genes and ion channel genes, with a minimum required combined score of 400. Shortest-path analysis identified bridge paths connecting hub genes to ion channel genes through at most two PPI intermediates (maximum path length of 4 nodes). Each bridge path received scoring based on the geometric mean of constituent edge scores. Evidence grading followed STRING evidence channels (experimental, database, textmining). Paths with at least one experimental or database evidence edge were graded as “strong”; others were graded as “moderate”.

Pathway enrichment analysis of druggable hub genes was performed using Enrichr [47,48] against the Reactome 2022 database. Enrichr computes enrichment using Fisher’s exact test with correction for multiple hypothesis testing, ranking gene sets by combined score (log of *p*-value multiplied by *z*-score of deviation from expected rank).

### 4.6. VGAE-Based Virtual Gene Knockout Validation

A virtual gene knockout pipeline was based on the GenKI methodology [20]. Two independent datasets provided input: single-cell RNA-seq count matrices for HCT116 cells downloaded from the Cell-omics Data Coordinate Platform (CDCP; dataset SCDS0000040, “Single-cell atlas of human cell lines”) and a single-cell RNA sequencing dataset (GSM5224587, HCT116 colorectal cancer cell line, mock condition from GSE171429).

Gene regulatory networks (GRNs) were constructed using k-nearest neighbor correlation graphs. The top 2000 most variable genes plus all genes involved in predicted bridge paths were included. A variational graph autoencoder (VGAE) was implemented using PyTorch 2.9.1 (used in VGAE implementation) with a two-layer graph convolutional network (GCN) [49] encoder and beta-VAE regularization. The VGAE architecture consists of the following:

**Encoder:** A two-layer GCN that maps node features (gene expression profiles) to a latent space:

$$h=\mathrm{ReLU}\left({\mathrm{GCN}}_{1}\left(X,A\right)\right),\;\mu ={\mathrm{GCN}}_{\mu }\left(h,A\right),\;\log\sigma ={\mathrm{GCN}}_{\sigma }\left(h,A\right)$$

where *X* is the gene expression matrix, *A* is the adjacency matrix, and latent embeddings *Z* are sampled via the reparameterization trick: Z=μ+ϵ⊙exp(logσ) with ϵ∼N(0,I).

**Decoder:** An inner product decoder reconstructs the adjacency matrix for link prediction (not expression value prediction):

$${\hat{A}}_{ij}=\sigma \left(z_{i}^{⊤}z_{j}\right)$$

where σ is the sigmoid function. The model predicts gene–gene co-expression relationships by reconstructing edges, not gene expression levels.

**Training objective:** The model minimizes the sum of reconstruction loss (binary cross-entropy on predicted edges) and KL divergence regularization:

$$L=L_{\mathrm{recon}}+L_{\mathrm{KL}}=-E\left[\log p\left(A|Z\right)\right]+\mathrm{KL}\left(q\left(Z|X,A\right)∥p\left(Z\right)\right)$$

Model training occurred once on the wild-type graph. The model was then frozen for all subsequent knockout inferences, preventing latent space misalignment between conditions.

**Virtual knockout mechanism:** We simulated gene knockout through a hybrid perturbation approach combining (1) feature zeroing (setting the target gene’s expression vector to zero) and (2) edge removal (removing all edges connected to the target gene), following the GenKI _KO_data_init procedure [20], ensuring complete removal of the gene’s contribution to the latent space. The frozen wild-type encoder was applied to the perturbed graph to obtain knockout latent representations $Z_{\mathrm{KO}}$. Per-gene KL divergence between knockout and wild-type latent distributions served as the perturbation signal:

$$\mathrm{KL}\left(Z_{\mathrm{KO}}∥Z_{\mathrm{WT}}\right)=\sum_{i=1}^{N}\mathrm{KL}\left(q\left(z_{i}^{\mathrm{KO}}\right)∥q\left(z_{i}^{\mathrm{WT}}\right)\right)$$

KL divergence values exhibited extreme right-skewness across 8+ orders of magnitude (range: $10^{-10}$ to $10^{7.6}$). Due to this extreme right-skewed distribution, we used percentile ranking rather than parametric statistics to quantify regulatory relationship strength. This non-parametric approach is robust to distributional assumptions and directly quantifies relative regulatory strength without requiring normality or homoskedasticity. For visualization, we report log_10_(KL + $10^{-10}$) in Supplementary Materials, preserving gene rankings while improving interpretability.

Significance testing was performed using GenKI bagging with bootstrap permutations. Permutation-based negative control analysis confirmed biological specificity: for each dataset, 50 randomly selected non-hub genes from the same gene set were subjected to virtual KO using the identical frozen VGAE model, and their KL divergence distributions served as the empirical null. This replaced the original bottom-10% expression control to avoid bias from low-abundance transcripts with inflated technical noise. Random seeds and control gene lists were saved alongside model weights for reproducibility. MAD-based *z*-scores were avoided due to their instability with heavy-tailed KL divergence distributions. Stability-based criteria prove more robust than magnitude-based tests for heavy-tailed distributions and align with the original GenKI methodology [20]. Bridge path validation required that the target ion channel or a path intermediate met the bagging criterion in at least one dataset.

### 4.7. Single-Cell Perturb-Seq (CRISPRi) Evidence Matrix: Data Source and Analysis Overview

As an independent experimental perturbation layer complementing the GenKI/VGAE-KO pipeline, an HCT116 CRISPRi Perturb-seq knockout dataset was obtained from the X-Atlas/Orion scalable Fix–Cryopreserve platform study [50]. CRISPRi (CRISPR interference) uses a catalytically dead Cas9 (dCas9) fused to a transcriptional repressor domain (KRAB) to silence target genes without DNA cleavage, as originally developed by Gilbert et al. [51]. The dataset comprises 8445 cells across 109 batches, with 6 knockout genes (*EXOSC5*, *LSM7*, *GALK1*, *RIPK2*, *TRMT112*, *RPS21*) across RNA processing, ribosomal, and metabolic programs based on their high network centrality scores and predicted ion channel bridge paths, plus non-targeting controls. Guide-level assignments were aggregated to gene-level knockout groups. A seven-strategy evidence matrix was used to assess hub gene → ion channel connections through complementary analytical approaches.

**Strategy 1 (Pseudobulk Differential Expression):** We performed batch-level pseudobulk aggregation to address zero inflation in low-expression ion channels. For each knockout gene, we aggregated cells by batch. We tested differential expression using pyDESeq2 [28] comparing knockout versus control batches. Pseudobulk aggregation proves particularly effective for lowly expressed genes where single-cell tests lack power. Genes with adjusted p<0.05 received scores of 3.0, p<0.1 received 2.0, p<0.2 received 1.0, otherwise 0.

**Strategy 2 (GSEA Pathway Enrichment):** Gene set enrichment analysis was performed using preranked GSEA on the knockout-induced transcriptome perturbation signature. Custom gene sets were constructed for ion channel families (K^+^ channels, Cl^−^ channels, glutamate receptors, etc.) and reactome pathways. Normalized enrichment scores (NES) with |NES| > 1.5 and p<0.05 received scores of 3.0, |NES| >1.3 received 2.0, |NES| > 1.0 received 1.0, otherwise 0. GSEA captures pathway-level effects that may not be detectable at the single-gene level.

**Strategy 3 (Transcriptome-wide Ranking):** Target ion channel genes were ranked by their perturbation magnitude (absolute log fold change) across the entire transcriptome. Genes ranking in the top 5% were scored 3.0; top 10% scored 2.0; top 20% scored 1.0; otherwise 0. Transcriptome-wide ranking identifies genes with the strongest knockout-induced expression changes relative to all other genes.

**Strategy 4 (Zero-Inflated Differential Expression):** Single-cell differential expression was performed using MAST (Model-based Analysis of Single-cell Transcriptomics), which explicitly models zero inflation through a two-part hurdle model separating detection rate (cellular detection rate, CDR) from expression level among detected cells. Genes with adjusted p<0.05 in either component were scored 3.0; p<0.1 scored 2.0; p<0.2 scored 1.0; otherwise 0.

**Strategy 5 (Indirect Regulatory Network):** For ion channels with minimal direct expression changes, we searched for indirect regulatory connections through differentially expressed intermediates. A gene was considered an indirect mediator if it was significantly perturbed ($p_{\mathrm{adj}}<0.05$) and had known functional associations (STRING database, combined score >400) with the target ion channel. Paths with validated intermediates received scores of 3.0, predicted intermediates 2.0, weak associations 1.0, no path 0.

**Strategy 6 (Global Perturbation Magnitude):** The overall transcriptome-wide perturbation effect was quantified as the mean absolute *z*-score across all genes following knockout. Global perturbation magnitude assessment determines whether the knockout produces a strong global perturbation signal, which may indicate successful gene silencing and downstream regulatory cascades. Mean |z|>0.5 received scores of 3.0, >0.3 received 2.0, >0.1 received 1.0, otherwise 0.

**Strategy 7 (Co-expression Disruption):** Pairwise Spearman correlation between the knockout gene and target ion channel was computed in control cells versus knockout cells. A substantial decrease in correlation (Δρ<−0.15) suggests disruption of co-expression structure, indicating functional coupling. |Δρ|>0.2 was scored 3.0; >0.15 scored 2.0; >0.1 scored 1.0; otherwise 0.

Each strategy was scored on a 0–3 scale, yielding a maximum total score of 21 per knockout gene → ion channel pair. The multi-strategy approach integrates orthogonal evidence types (pseudobulk versus single-cell, direct versus indirect, expression versus pathway, correlation versus perturbation magnitude) to provide reliable validation that does not rely on a single statistical test. Complete strategy scores are provided in Table S5.

**Composite validation score.** To integrate evidence from both validation modalities, we computed a composite validation score as a weighted average: Composite Score = 0.6 × (Perturb-seq score/21) + 0.4 × (average VGAE-KO percentile/100). Perturb-seq received higher weight (60%) as it represents direct experimental validation, while VGAE-KO (40%) provides computational support through virtual knockout simulations. This weighting balances experimental evidence with in silico prediction, prioritizing empirical validation while adding computational insights. The composite score ranges from 0 to 1, with higher values indicating stronger convergent evidence across both modalities.

## 5. Conclusions

The integrated pipeline identified hub genes across three functional programs in CRC. These genes connect to ion channels across 7 families via PPI-mediated bridge paths. Among 100 hub genes from WGCNA, 23 had ion-channel bridge paths and 11 were druggable. Eight carry drugs suitable for cancer repurposing after excluding three targets: two immunosuppressive agents (*ITGAL*, *CD6*) and one hub gene that promotes a tumor suppressor ion channel (*RIPK2* → *CFTR*). Ribosomal proteins link to K^+^ channels. *RPS21* → *KCNQ2* passed dual perturbation validation and can be targeted with EMA-approved ataluren. RNA processing genes connect to Cl^−^ channels. *LSM7* → *CLIC1* showed the strongest concordant validation (Perturb-seq 15.5/21; VGAE-KO: 99.8th and 99.4th percentile, 100% bootstrap consistency) and represents the most promising target for future drug development given *CLIC1*’s established pro-tumorigenic role in CRC [14,15] and high tractability scores despite the absence of approved inhibitors. Immune checkpoint receptors connect via PPI intermediates to Ca^2+^ and K^+^ channels, targetable by relatlimab (FDA-approved; immune-enhancing) and varlilumab (Phase 2; immune-enhancing). These need validation in immunocompetent models. These findings reveal previously uncharacterized connections between CRC driver genes and ion channel regulation, supporting rational repurposing strategies applicable to other cancer types.

## Figures and Tables

**Table 1:** **Top 10 Hub Genes Ranked by Composite Score.** Module membership (MM), intramodular connectivity (kWithin), gene significance (GS), and composite score for the highest-ranked hub genes from WGCNA co-expression network analysis.

Gene	Module	MM	kWithin	GS	Score	Direction	Function
*EXOSC5*	green	0.399	87.7	0.244	0.804	Up	RNA exosome
*LAGE3*	green	0.473	86.8	0.213	0.789	Up	tRNA modification
*NAA10*	green	0.425	75.5	0.233	0.750	Up	N-acetyltransferase
*PDCD5*	green	0.462	94.4	0.161	0.745	Up	Apoptosis
*SNHG6*	green	0.538	93.8	0.124	0.723	Up	lncRNA
*SNRPD2*	green	0.510	79.0	0.176	0.721	Up	Spliceosome
*TRMT112*	green	0.536	89.4	0.120	0.698	Up	tRNA methyltransferase
*LSM7*	green	0.439	75.7	0.180	0.683	Up	RNA processing
*CCDC167*	green	0.536	61.9	0.180	0.666	Up	Coiled-coil protein
*NT5C3B*	green	0.529	58.4	0.190	0.662	Up	Nucleotidase

**Table 2:** **Druggable Hub Genes with Drug Evidence.** Drug targets cataloged through OpenTargets, DGIdb, and ChEMBL mining. Phase: maximum clinical development phase (4 = FDA-approved). Score: composite DrugEvidenceScore. ^†^ Immunosuppressive (excluded). ^‡^ Immune-enhancing: retained as repurposing candidates. * *CFTR* is a tumor suppressor in intestinal cancer [13]; hub genes promoting *CFTR* are excluded from repurposing candidacy as *CFTR* inhibition would be therapeutically counterproductive.

Gene	Program	Phase	Representative Drug	Score	Ion Channel
*ITGAL*	Immune ^†^	4	Efalizumab, Lifitegrast	0.919	*GRIN2A*
*RPS19*	Ribosomal	4	Ataluren, ELX-02	0.865	*KCNA10*
*RPS2*	Ribosomal	4	Ataluren	0.835	*GRIN2B*
*RPS21*	Ribosomal	4	Ataluren	0.828	*KCNQ2*
*RPL12*	Ribosomal	4	Ataluren	0.828	*KCNA10*
*RPL39*	Ribosomal	4	Ataluren	0.828	*GRIN2B*
*LAG3*	Immune ^‡^	4	Relatlimab, Fianlimab	0.778	*RYR3*
*CD6*	Immune ^†^	3	Itolizumab	0.705	*GRIK2*
*CD27*	Immune ^‡^	2	Varlilumab	0.611	*KCNN3*
*GALK1*	Metabolic	–	Quercetin (DGIdb)	0.362	*KCNA5*
*RIPK2*	RNA processing	–	Ponatinib (DGIdb)	0.355	*CFTR* *

**Table 3:** **Ion Channel Bridge Paths with STRING Evidence.** Hub gene → ion channel connections via PPI intermediates. All individual edges verified in STRING v12.0 (combined score ≥ 400). Score: path score computed as the geometric mean of constituent edge scores. Evidence grade: strong = experimental/database support; moderate = textmining only. ^†^ Immune-suppressive drug: excluded from repurposing. ^‡^ Immune-enhancing drug: retained as repurposing candidate. * *CFTR* is a tumor suppressor in intestinal cancer [13]; hub genes promoting *CFTR* are excluded from repurposing candidacy as *CFTR* inhibition would be therapeutically counterproductive.

Hub Gene	PPI Path	Ion Channel	Score	Grade	Drug
*Ribosomal program*					
*RPS21*	*RPS21* → *KCNQ2*	*KCNQ2* (K^+^)	0.666	strong	Ataluren
*RPS2*	*RPS2* → *RACK1* → *GRIN2B*	*GRIN2B* (Glu)	0.559	strong	Ataluren
*RPL39*	*RPL39* → *RACK1* → *GRIN2B*	*GRIN2B* (Glu)	0.507	strong	Ataluren
*RPS19*	*RPS19* → *KCNA10*	*KCNA10* (K^+^)	0.676	strong	Ataluren
*RPL12*	*RPL12* → *KCNA10*	*KCNA10* (K^+^)	0.539	strong	Ataluren
*RNA processing program*					
*LSM7*	*LSM7* → *LSM1* → *CLIC1*	*CLIC1* (Cl^−^)	0.560	strong	–
*RIPK2* *	*RIPK2* → *HSPA8* → *CFTR*	*CFTR* * (Cl^−^)	0.436	strong	Ponatinib
*TRMT112* *	*TRMT112* → *RPS27A* → *CFTR*	*CFTR* * (Cl^−^)	0.428	strong	–
*EXOSC5*	*EXOSC5* → *EXOSC8* → *AQP9*	*AQP9* (H_2_O)	0.532	strong	–
*NAA10*	*NAA10* → *GRIN1*	*GRIN1* (Glu)	0.495	moderate	–
*SNRPD2*	*SNRPD2* → *SNRPN* → *GABRB3*	*GABRB3* (GABA)	0.555	strong	–
*Metabolic program*					
*GALK1*	*GALK1* → *TPI1* → *KCNA5*	*KCNA5* (K^+^)	0.279	moderate	Quercetin
*Immune program*					
*ITGAL* ^†^	*ITGAL* → *SRC* → *GRIN2A*	*GRIN2A* (Glu)	0.519	strong	–
*LAG3* ^‡^	*LAG3* → *CD38* → *RYR3*	*RYR3* (RyR)	0.421	strong	Relatlimab
*CD6* ^†^	*CD6* → *SDCBP* → *GRIK2*	*GRIK2* (Glu)	0.432	strong	–
*CD27* ^‡^	*CD27* → *KCNN3*	*KCNN3* (K^+^)	0.586	moderate	Varlilumab
*FCRL5*	*FCRL5* → *CD38* → *RYR3*	*RYR3* (RyR)	0.396	strong	–
*APBB1IP*	*APBB1IP* → *SRC* → *KCNA5*	*KCNA5* (K^+^)	0.413	strong	–
*Other*					
*LAGE3*	*LAGE3* → *TP53RK* → *ANO4*	*ANO4* (Ano)	0.454	strong	–
*PDCD5*	*PDCD5* → *KAT5* → *TRPC3*	*TRPC3* (TRP)	0.482	strong	–
*PFDN4*	*PFDN4* → *VBP1* → *CLIC2*	*CLIC2* (Cl^−^)	0.427	strong	–
*S100A2*	*S100A2* → *S100A1* → *RYR2*	*RYR2* (RyR)	0.445	strong	–
*CCDC167*	*CCDC167* → *PRKCSH* → *PKD2*	*PKD2* (Poly)	0.277	moderate	–

**Table 4:** **VGAE-KO Validation Summary.** Virtual gene knockout validation results for hub gene → ion channel pairs across two independent scRNA-seq datasets. Percentile: (1 − Rank/Total) × 100%. Bootstrap: percentage of 100 permutations where gene was ranked in top 5%. Status: VALIDATED = target channel was directly validated; PARTIAL = path intermediate was validated.

KO Gene	Ion Channel	Percentile		Bootstrap		Concordant	Status	Validated Gene
		SCDS	GSM	SCDS	GSM			
*LSM7*	*CLIC1*	99.8%	99.4%	100%	100%	Yes	VALIDATED	*CLIC1*
*RPS21*	*KCNQ2*	97.8%	98.7%	100%	100%	Yes	VALIDATED	*KCNQ2*
*EXOSC5*	*AQP9*	97.6%	98.8%	100%	100%	Yes	PARTIAL	*EXOSC8*
*GALK1*	*KCNA5*	99.9%	99.6%	100%	100%	Yes	PARTIAL	*TPI1*
*RIPK2*	*CFTR*	99.9%	3.0%	100%	0%	No	PARTIAL	*HSPA8* (SCDS)
*TRMT112*	*CFTR*	99.7%	6.4%	100%	0%	No	PARTIAL	*RPS27A* (SCDS)
*RPL39*	*GRIN2B*	76.5%	98.5%	0%	100%	Yes	VALIDATED	*GRIN2B*
*RPS2*	*GRIN2B*	54.2%	98.5%	0%	100%	No	VALIDATED	*GRIN2B* (GSM)

SCDS: SCDS0000040 dataset (HCT116, Cell-omics Data Coordinate Platform); GSM: GSM5224587 dataset (HCT116, GEO). Percentile: (1 − Rank/Total) × 100%. Bootstrap: percentage of 100 permutations where gene was ranked in top 5%.

**Table 5:** **HCT116 Perturb-seq Multi-Strategy Evidence Matrix.** Integrated evidence scores from seven complementary validation strategies for six KO gene → ion channel pairs. S1: Pseudobulk DE; S2: GSEA; S3: Ranking; S4: MAST; S5: Network; S6: Perturbation; S7: Co-expression. Maximum score per strategy: 3.

KO → Target	S1	S2	S3	S4	S5	S6	S7	Total
*LSM7* → *CLIC1*	1.0	3.0	3.0	1.5	3.0	2.0	2.0	15.5
*RPS21* → *KCNQ2*	1.5	0.5	2.0	0.5	1.5	0.5	2.0	8.5
*RIPK2* → *CFTR*	2.0	1.0	0.5	1.5	1.5	0.5	0.5	7.5
*GALK1* → *KCNA5*	0.5	2.0	1.0	1.5	0.3	0.5	0.5	6.3
*TRMT112* → *CFTR*	2.0	0.5	0.5	0.5	0.3	0.5	0.5	4.8
*EXOSC5* → *AQP9*	0.5	0.5	0.5	0.5	0.3	0.5	0.5	3.3

## Data Availability

All datasets are publicly available: (1) **Discovery Cohorts (Bulk RNA-seq):** GSE196006 (*n* = 42): https://www.ncbi.nlm.nih.gov/geo/query/acc.cgi?acc=GSE196006 (accessed on 4 April 2026); GSE251845 (*n* = 43): https://www.ncbi.nlm.nih.gov/geo/query/acc.cgi?acc=GSE251845 (accessed on 4 April 2026). (2) **External Validation Cohorts (Bulk RNA-seq):** GSE128969 (*n* = 6): https://www.ncbi.nlm.nih.gov/geo/query/acc.cgi?acc=GSE128969 (accessed on 4 April 2026); GSE138202 (*n* = 16): https://www.ncbi.nlm.nih.gov/geo/query/acc.cgi?acc=GSE138202 (accessed on 4 April 2026); GSE95132 (*n* = 24): https://www.ncbi.nlm.nih.gov/geo/query/acc.cgi?acc=GSE95132 (accessed on 4 April 2026). (3) **Single-Cell RNA-seq (VGAE-KO Validation):** SCDS0000040: https://ngdc.cncb.ac.cn/gsa/browse/CRA004659 (accessed on 4 April 2026); GSM5224587: https://www.ncbi.nlm.nih.gov/geo/query/acc.cgi?acc=GSE171429 (accessed on 4 April 2026). (4) **Perturb-seq Data (Experimental Validation):** HCT116 CRISPRi Perturb-seq (Replogle et al., Cell 2022): https://plus.figshare.com/ndownloader/files/55021257 (accessed on 4 April 2026). (5) **External Databases:** STRING v12.0: https://string-db.org/api/json/network (accessed on 4 April 2026); OpenTargets: https://api.platform.opentargets.org/api/v4/graphql (accessed on 4 April 2026); DGIdb: https://dgidb.org/api/ (accessed on 4 April 2026); HGNC: https://rest.genenames.org/fetch/symbol/\{symbol\} (accessed on 4 April 2026); ChEMBL Target Search: https://www.ebi.ac.uk/chembl/api/data/target/search.json (accessed on 4 April 2026); ChEMBL Mechanism: https://www.ebi.ac.uk/chembl/api/data/mechanism.json (accessed on 4 April 2026). All analysis code and processed data are available at https://github.com/dong-zhongyuan/crc-ion-channel-repurposing (accessed on 4 April 2026). The repository includes trained VGAE model weights (.pt files), negative control random seeds (.json), pre-processed Perturb-seq count matrices, and all intermediate analysis outputs required for full reproducibility.

## Supplementary Materials

The following supporting information can be downloaded at: https://www.mdpi.com/article/10.3390/ijms27083412/s1.

## Abbreviations

The following abbreviations are used in this manuscript:

AUC	Area Under the Curve
CRC	Colorectal Cancer
CRISPRi	CRISPR interference
DEG	Differentially Expressed Gene
FDA	Food and Drug Administration
FDR	False Discovery Rate
GEO	Gene Expression Omnibus
GO	Gene Ontology
KEGG	Kyoto Encyclopedia of Genes and Genomes
KL	Kullback–Leibler
KO	Knockout
NGDC	National Genomics Data Center
PCA	Principal Component Analysis
PPI	Protein–Protein Interaction
RNA-seq	RNA Sequencing
ROC	Receiver Operating Characteristic
scRNA-seq	Single-cell RNA Sequencing
VGAE	Variational Graph Autoencoder
WGCNA	Weighted Gene Co-expression Network Analysis
