# Supplementary material for: Ion-Channel-Mediated Drug Repurposing Opportunities Validated by Single-Cell Perturbation in Colorectal Cancer
Source: Int J Mol Sci. 2026 Apr 10;27(8):3412. doi: 10.3390/ijms27083412 (PMC13116841; doi:10.3390/ijms27083412)
Supplement: Supplementary file 1 [file ijms-27-03412-s001.zip › ijms-4224662-supplementary.pdf]

Supplementary Tables

Supplementary Table S1

Complete Hub Gene Candidate List (219 genes; top 100 selected as hub genes). GS = gene significance, MM = module membership, kWithin = intramodular connectivity. All metrics normalized to 0–1 scale.

Gene	Module	GS	MM	kWithin	Composite	Direction	Rank
EXOSC5	green	1.000	0.496	0.917	0.804	Up	1
LAGE3	green	0.873	0.589	0.906	0.789	Up	2
NAA10	green	0.955	0.529	0.764	0.750	Up	3
PDCD5	green	0.659	0.575	1.000	0.745	Up	4
SNHG6	green	0.508	0.670	0.992	0.723	Up	5
SNRPD2	green	0.721	0.635	0.807	0.721	Up	6
TRMT112	green	0.490	0.666	0.937	0.698	Up	7
LSM7	green	0.738	0.546	0.766	0.683	Up	8
CCDC167	green	0.737	0.666	0.594	0.666	Up	9
NT5C3B	green	0.779	0.658	0.550	0.662	Up	10
RPS19	green	0.653	0.656	0.652	0.654	Up	11
IGHV3-21	red	0.046	0.914	1.000	0.653	Up	12
RPS2	green	0.695	0.533	0.719	0.649	Up	13
IGHV4-59	red	0.119	0.949	0.844	0.637	Up	14
PSMG4	green	0.799	0.535	0.568	0.634	Up	15
IGHV3-15	red	0.067	0.924	0.885	0.626	Up	16
IGHV3-74	red	0.075	0.950	0.847	0.624	Up	17
RPS21	green	0.531	0.599	0.741	0.624	Up	18
RPL12	green	0.496	0.680	0.677	0.618	Up	19
ZFAS1	green	0.581	0.577	0.695	0.617	Up	20
GAS5	green	0.559	0.661	0.628	0.616	Up	21
METTL26	green	0.592	0.593	0.651	0.612	Up	22
IGHV3-73	red	0.332	0.825	0.672	0.610	Up	23
GALK1	green	0.763	0.505	0.550	0.606	Up	24
NOP56	green	0.708	0.504	0.600	0.604	Up	25
PFDN4	green	0.527	0.528	0.647	0.567	Up	26
SNHG5	green	0.731	0.592	0.358	0.560	Up	27
FCRL5	red	0.052	0.834	0.783	0.556	Up	28
MRPS26	green	0.772	0.538	0.318	0.543	Up	29
IGHV6-1	red	0.253	0.851	0.514	0.539	Up	30
FXYD5	green	0.697	0.468	0.446	0.537	Up	31
RIPK2	green	0.398	0.427	0.771	0.532	Up	32
RPL39	green	0.589	0.556	0.450	0.532	Up	33
RPL36AP37	green	0.511	0.585	0.493	0.530	Up	34
IGHV3-53	red	0.113	0.867	0.593	0.525	Up	35
IGHV3-43	red	0.412	0.717	0.438	0.522	Up	36
NXT1	green	0.485	0.485	0.573	0.514	Up	37
IGHV4-34	red	0.088	0.838	0.595	0.507	Up	38
IGHV1-18	red	0.009	0.885	0.626	0.507	Up	39
DUSP14	green	0.425	0.541	0.549	0.505	Up	40
MAD2L2	green	0.526	0.644	0.333	0.501	Up	41
IGKV2-24	red	0.036	1.000	0.464	0.500	Up	42

Continued on next page

Supplementary Table S1 (continued)

**Gene**	**Module**	**GS**	**MM**	**kWithin**	**Composite**	**Direction**	**Rank**
LINC01315	green	0.485	0.494	0.520	0.500	Up	43
CD27	red	0.076	0.793	0.622	0.497	Up	44
IGKV3D-15	red	0.216	0.757	0.502	0.492	Up	45
IGLV3-19	red	0.006	0.925	0.538	0.490	Up	46
IGHV3-49	red	0.178	0.779	0.507	0.488	Up	47
POU2AF1	red	0.170	0.804	0.485	0.486	Up	48
IGHV1-69D	red	0.266	0.767	0.406	0.480	Up	49
IGHV3-66	red	0.237	0.720	0.469	0.475	Up	50
IGLV5-45	red	0.190	0.839	0.378	0.469	Up	51
IGKV2D-29	red	0.185	0.772	0.430	0.462	Up	52
ARHGAP30	pink	0.017	0.452	0.899	0.456	Up	53
IGHV4-28	red	0.133	0.730	0.503	0.455	Up	54
HCLS1	pink	0.027	0.493	0.835	0.452	Up	55
SLC27A5	green	0.384	0.624	0.344	0.451	Up	56
MYO1G	pink	0.240	0.430	0.668	0.446	Up	57
ARHGAP25	pink	0.005	0.328	1.000	0.444	Up	58
FAM83E	green	0.029	0.825	0.450	0.435	Up	59
IGHV2-26	red	0.162	0.688	0.440	0.430	Up	60
IFT22	green	0.110	0.667	0.511	0.429	Up	61
IGLV2-18	red	0.066	0.832	0.386	0.428	Up	62
APBB1IP	pink	0.095	0.434	0.755	0.428	Up	63
S100A2	green	0.391	0.438	0.444	0.424	Up	64
NEURL1	green	0.353	0.564	0.330	0.416	Up	65
IGHV4-61	red	0.106	0.713	0.425	0.415	Up	66
IGHV1-69	red	0.353	0.615	0.268	0.412	Up	67
FMNL1	pink	0.427	0.349	0.458	0.411	Up	68
IGHV4-4	red	0.268	0.746	0.216	0.410	Up	69
IGHV2-70	red	0.450	0.598	0.171	0.406	Up	70
RASSF5	pink	0.120	0.376	0.695	0.397	Up	71
IGHV2-70D	red	0.484	0.568	0.138	0.397	Up	72
IGHV3-20	red	0.138	0.674	0.373	0.395	Up	73
IGHV4-31	red	0.071	0.725	0.381	0.392	Up	74
IGHV1-24	red	0.309	0.641	0.224	0.391	Up	75
AGTRAP	green	0.262	0.587	0.322	0.390	Up	76
WAS	pink	0.050	0.425	0.679	0.385	Up	77
LAX1	red	0.064	0.692	0.399	0.385	Up	78
NMB	green	0.122	0.556	0.476	0.385	Up	79
IGHV3-13	red	0.084	0.719	0.341	0.381	Up	80
RASAL3	pink	0.300	0.126	0.717	0.381	Up	81
MYO1F	pink	0.222	0.521	0.399	0.381	Up	82
IGHV1-58	red	0.552	0.514	0.070	0.379	Up	83
IFITM3	green	0.282	0.522	0.332	0.379	Up	84
IGKV6-21	red	0.038	0.794	0.300	0.377	Up	85
IGKV1-8	red	0.022	0.753	0.346	0.374	Up	86
TNFSF8	pink	0.276	0.400	0.442	0.373	Up	87
LAG3	pink	0.510	0.244	0.355	0.369	Up	88

Continued on next page

Supplementary Table S1 (continued)

Gene	Module	GS	MM	kWithin	Composite	Direction	Rank
POMP	green	0.555	0.291	0.258	0.368	Up	89
FAM30A	red	0.006	0.777	0.317	0.367	Up	90
CD6	pink	0.663	0.115	0.316	0.365	Up	91
FRYL	green	0.084	0.734	0.272	0.363	Up	92
CMTM7	green	0.200	0.574	0.311	0.362	Up	93
IGHV1-3	red	0.336	0.682	0.063	0.360	Up	94
FAM78A	pink	0.050	0.224	0.798	0.357	Up	95
EVL	pink	0.206	0.198	0.661	0.355	Up	96
ITGAL	pink	0.068	0.199	0.794	0.353	Up	97
AGFG2	green	0.146	0.566	0.348	0.353	Up	98
CARMIL2	pink	0.511	0.199	0.348	0.353	Up	99
TNFAIP2	pink	0.321	0.412	0.325	0.353	Up	100
IGKV2D-24	red	0.272	0.650	0.134	0.352	Up	101
ACAP1	pink	0.467	0.078	0.509	0.352	Up	102
PLCB2	pink	0.335	0.357	0.357	0.350	Up	103
ARHGAP9	pink	0.067	0.268	0.713	0.349	Up	104
CD3E	pink	0.250	0.147	0.639	0.345	Up	105
THEMIS2	pink	0.277	0.524	0.226	0.342	Up	106
SH3BP1	green	0.187	0.587	0.244	0.339	Up	107
LCK	pink	0.810	0.067	0.130	0.336	Up	108
GNE	green	0.110	0.663	0.230	0.335	Up	109
SIPA1L2	green	0.061	0.588	0.346	0.332	Up	110
PREX1	pink	0.204	0.444	0.346	0.331	Up	111
MIDN	green	0.104	0.681	0.206	0.330	Up	112
ZAP70	pink	0.401	0.000	0.587	0.330	Up	113
RHOH	pink	0.325	0.357	0.303	0.329	Up	114
DOK3	pink	0.048	0.369	0.568	0.329	Up	115
SPOCK2	pink	0.429	0.153	0.400	0.327	Up	116
CHST11	pink	0.092	0.506	0.376	0.325	Up	117
IL21R	pink	0.624	0.080	0.269	0.324	Up	118
MAP4K1	pink	0.154	0.098	0.708	0.320	Up	119
RPL39P3	green	0.234	0.415	0.311	0.320	Up	120
IGLV1-36	red	0.153	0.638	0.167	0.319	Up	121
PLCG2	pink	0.001	0.358	0.596	0.318	Up	122
WIPF1	pink	0.118	0.411	0.426	0.318	Up	123
MIAT	pink	0.705	0.041	0.208	0.318	Up	124
FYN	pink	0.507	0.309	0.131	0.316	Up	125
TAGAP	pink	0.053	0.499	0.384	0.312	Up	126
NLRC3	pink	0.444	0.106	0.383	0.311	Up	127
LINC00861	pink	0.365	0.330	0.235	0.310	Up	128
FERMT3	pink	0.131	0.482	0.315	0.309	Up	129
ELMO1	pink	0.150	0.454	0.322	0.309	Up	130
SLC25A25-AS1	green	0.039	0.586	0.294	0.306	Up	131
JAK3	pink	0.807	0.007	0.098	0.304	Up	132
IFI16	pink	0.124	0.586	0.201	0.304	Up	133
P2RY8	pink	0.150	0.207	0.553	0.304	Up	134

Continued on next page

Supplementary Table S1 (continued)

Gene	Module	GS	MM	kWithin	Composite	Direction	Rank
TPSG1	green	0.109	0.484	0.316	0.303	Up	135
SLA	pink	0.002	0.482	0.411	0.298	Up	136
IGLC7	red	0.204	0.688	0.000	0.297	Up	137
PPP1R16B	pink	0.029	0.242	0.619	0.296	Up	138
IGLV9-49	red	0.117	0.596	0.172	0.295	Up	139
PIK3CD	pink	0.024	0.237	0.625	0.295	Up	140
RUNX3	pink	0.206	0.344	0.328	0.292	Up	141
TBC1D10C	pink	0.204	0.011	0.661	0.292	Up	142
CHST2	pink	0.256	0.445	0.169	0.290	Up	143
BHLHA15	red	0.373	0.435	0.061	0.290	Up	144
SPN	pink	0.148	0.338	0.380	0.289	Up	145
ARHGAP4	pink	0.443	0.236	0.187	0.289	Up	146
CLEC2D	pink	0.435	0.061	0.368	0.288	Up	147
NUGGC	red	0.096	0.636	0.131	0.288	Up	148
SIRPG	pink	0.464	0.041	0.333	0.279	Up	149
IGKV2-29	red	0.286	0.446	0.100	0.277	Up	150
MMP25	pink	0.236	0.394	0.189	0.273	Up	151
ARL4C	pink	0.117	0.638	0.062	0.273	Up	152
IL18R1	pink	0.289	0.425	0.103	0.272	Up	153
PROM2	green	0.042	0.479	0.292	0.271	Up	154
IL7R	pink	0.272	0.426	0.114	0.271	Up	155
PF4	green	0.077	0.447	0.286	0.270	Up	156
CORO1A	pink	0.105	0.183	0.520	0.269	Up	157
ITGB2-AS1	pink	0.444	0.104	0.260	0.269	Up	158
RAC2	pink	0.279	0.223	0.297	0.266	Up	159
CHI3L2	pink	0.265	0.309	0.219	0.265	Up	160
DAPK2	green	0.000	0.591	0.196	0.262	Up	161
CXCR4	pink	0.218	0.291	0.273	0.261	Up	162
ITK	pink	0.088	0.229	0.462	0.259	Up	163
STX11	pink	0.059	0.508	0.203	0.257	Up	164
IGKV1D-13	red	0.053	0.552	0.154	0.253	Up	165
SEPTIN1	pink	0.193	0.043	0.518	0.252	Up	166
SOCS1	pink	0.572	0.100	0.080	0.251	Up	167
CCL5	pink	0.249	0.267	0.235	0.250	Up	168
IL2RB	pink	0.019	0.349	0.369	0.246	Up	169
LIMD2	pink	0.304	0.015	0.412	0.244	Up	170
GPR171	pink	0.197	0.302	0.228	0.243	Up	171
SP140	pink	0.044	0.177	0.505	0.242	Up	172
IGKV2D-40	red	0.167	0.459	0.098	0.241	Up	173
ETS1	pink	0.036	0.390	0.298	0.241	Up	174
TYMP	pink	0.016	0.543	0.162	0.240	Up	175
CCR7	pink	0.427	0.008	0.285	0.240	Up	176
LTB	pink	0.319	0.070	0.328	0.239	Up	177
SMAP2	pink	0.117	0.397	0.200	0.238	Up	178
CYTIP	pink	0.075	0.401	0.233	0.236	Up	179
MIR142HG	pink	0.314	0.304	0.088	0.235	Up	180

Continued on next page

Supplementary Table S1 (continued)

Gene	Module	GS	MM	kWithin	Composite	Direction	Rank
POU2F2	pink	0.223	0.079	0.395	0.232	Up	181
RFTN1	pink	0.075	0.278	0.339	0.231	Up	182
CD247	pink	0.133	0.117	0.440	0.230	Up	183
SLAMF1	pink	0.019	0.328	0.327	0.225	Up	184
JAML	pink	0.048	0.483	0.142	0.224	Up	185
GRAP2	pink	0.180	0.100	0.392	0.224	Up	186
KLK3	green	0.211	0.355	0.102	0.223	Up	187
CHRM3-AS2	pink	0.272	0.149	0.243	0.221	Up	188
PILRA	pink	0.050	0.613	0.000	0.221	Up	189
PTPN7	pink	0.398	0.049	0.212	0.220	Up	190
IGKV5-2	red	0.022	0.546	0.085	0.218	Up	191
TMC8	pink	0.324	0.144	0.179	0.216	Up	192
CIITA	pink	0.109	0.213	0.316	0.213	Up	193
TIGIT	pink	0.222	0.038	0.372	0.211	Up	194
CD28	pink	0.044	0.136	0.451	0.210	Up	195
SIT1	pink	0.266	0.133	0.228	0.209	Up	196
CCR4	pink	0.358	0.165	0.101	0.208	Up	197
UCP2	pink	0.046	0.385	0.187	0.206	Up	198
IKZF3	pink	0.129	0.039	0.432	0.200	Up	199
CD2	pink	0.126	0.052	0.407	0.195	Up	200
PARP15	pink	0.257	0.033	0.294	0.195	Up	201
SELL	pink	0.182	0.230	0.172	0.195	Up	202
IPCEF1	pink	0.135	0.100	0.337	0.191	Up	203
TRAC	pink	0.042	0.066	0.457	0.189	Up	204
TRBC1	pink	0.182	0.049	0.309	0.180	Up	205
RAB26	green	0.307	0.222	0.000	0.176	Up	206
SH2D1A	pink	0.215	0.017	0.291	0.175	Up	207
SH2D3C	pink	0.079	0.169	0.266	0.171	Up	208
PRF1	pink	0.013	0.224	0.253	0.163	Up	209
SLAMF6	pink	0.182	0.041	0.215	0.146	Up	210
PLA2G2D	pink	0.047	0.095	0.284	0.142	Up	211
CD72	pink	0.066	0.134	0.223	0.141	Up	212
TRBC2	pink	0.020	0.049	0.339	0.136	Up	213
APOL3	pink	0.160	0.137	0.107	0.135	Up	214
S1PR4	pink	0.331	0.025	0.039	0.132	Up	215
CD40	pink	0.020	0.242	0.079	0.113	Up	216
CD3D	pink	0.057	0.063	0.195	0.105	Up	217
CXCR2P1	pink	0.098	0.126	0.077	0.100	Up	218
DTX1	pink	0.087	0.002	0.190	0.093	Up	219

Supplementary Table S2

Complete Hub Gene → Ion Channel Bridge Paths (23 paths). PPI-mediated connections via STRING v12.0 (combined score ≥ 400).

Hub Gene	Path	Ion Channel	Length	Score
APBB1IP	→*SRC*→*KCNA5*	*KCNA5*	2	0.41
CD27	→*KCNN3*	*KCNN3*	1	0.59
CCDC167	→*PRKCSH*→*PKD2*	*PKD2*	2	0.28
CD6	→*SDCBP*→*GRIK2*	*GRIK2*	2	0.43
EXOSC5	→*EXOSC8*→*AQP9*	*AQP9*	2	0.53
FCRL5	→*CD38*→*RYR3*	*RYR3*	2	0.40
GALK1	→*TPI1*→*KCNA5*	*KCNA5*	2	0.28
ITGAL	→*SRC*→*GRIN2A*	*GRIN2A*	2	0.52
LAG3	→*CD38*→*RYR3*	*RYR3*	2	0.42
LAGE3	→*TP53RK*→*ANO4*	*ANO4*	2	0.45
NAA10	→*GRIN1*	*GRIN1*	1	0.50
LSM7	→*LSM1*→*CLIC1*	*CLIC1*	2	0.56
PDCD5	→*KAT5*→*TRPC3*	*TRPC3*	2	0.48
PFDN4	→*VBP1*→*CLIC2*	*CLIC2*	2	0.43
RIPK2	→*HSPA8*→*CFTR*	*CFTR*	2	0.44
RPL12	→*KCNA10*	*KCNA10*	1	0.54
RPL39	→*RACK1*→*GRIN2B*	*GRIN2B*	2	0.51
RPS19	→*KCNA10*	*KCNA10*	1	0.68
RPS2	→*RACK1*→*GRIN2B*	*GRIN2B*	2	0.56
RPS21	→*KCNQ2*	*KCNQ2*	1	0.666
S100A2	→*S100A1*→*RYR2*	*RYR2*	2	0.44
SNRPD2	→*SNRPN*→*GABRB3*	*GABRB3*	2	0.56
TRMT112	→*RPS27A*→*CFTR*	*CFTR*	2	0.43

Supplementary Table S3

Druggable Hub Genes with Drug Evidence. Hub Gene Druggability Assessment. This table includes all 67 hub genes (of 100) with non-zero druggability scores. Genes with Phase > 0 or DGIdb evidence have actual drug associations; others represent computational druggability predictions. DrugEvidenceScore = 0.50 × Phase + 0.25 × Tractability + 0.15 × DGIdb + 0.10 × ChEMBL. Phase 4 = clinically approved (FDA or EMA).

Gene	Phase	Score	Approved	Representative Drugs
ITGAL	4	0.919	10	EFALIZUMAB,
RPS19	4	0.865	1	ATALUREN, ELX
RPS2	4	0.835	1	ATALUREN, ELX
RPS21	4	0.828	1	ATALUREN, ELX
RPL12	4	0.828	1	ATALUREN, ELX
RPL39	4	0.828	1	ATALUREN, ELX
LAG3	4	0.903	1	RELATLIMAB,
CD6	3	0.705	0	ITOLIZUMAB
CD27	2	0.611	0	VARLILUMAB
GALK1	0	0.362	0	
RIPK2	0	0.355	0	
S100A2	0	0.298	0	
PFDN4	0	0.280	0	
APBB1IP	0	0.280	0	
FCRL5	0	0.270	0	
EXOSC5	0	0.250	0	
LAGE3	0	0.250	0	
NAA10	0	0.250	0	
PDCD5	0	0.250	0	
SNRPD2	0	0.250	0	
TRMT112	0	0.250	0	
LSM7	0	0.250	0	
CCDC167	0	0.250	0	
NT5C3B	0	0.250	0	
IGHV4-59	0	0.250	0	
PSMG4	0	0.250	0	
METTL26	0	0.250	0	
NOP56	0	0.250	0	
MRPS26	0	0.250	0	
FXYD5	0	0.250	0	
IGHV3-53	0	0.250	0	
NXT1	0	0.250	0	
IGHV4-34	0	0.250	0	
DUSP14	0	0.250	0	
MAD2L2	0	0.250	0	
IGLV3-19	0	0.250	0	
POU2AF1	0	0.250	0	
ARHGAP30	0	0.250	0	
HCLS1	0	0.250	0	
SLC27A5	0	0.250	0	

Continued on next page

Supplementary Table S3 (continued)

Gene	Phase	Score	Approved	Representative Drugs
MYO1G	0	0.250	0	
ARHGAP25	0	0.250	0	
IFT22	0	0.250	0	
NEURL1	0	0.250	0	
IGHV1-69	0	0.250	0	
FMNL1	0	0.250	0	
IGHV2-70	0	0.250	0	
RASSF5	0	0.250	0	
AGTRAP	0	0.250	0	
WAS	0	0.250	0	
LAX1	0	0.250	0	
NMB	0	0.250	0	
IGHV3-13	0	0.250	0	
RASAL3	0	0.250	0	
MYO1F	0	0.250	0	
IFITM3	0	0.250	0	
TNFSF8	0	0.250	0	
POMP	0	0.250	0	
FRYL	0	0.250	0	
CMTM7	0	0.250	0	
FAM78A	0	0.250	0	
EVL	0	0.250	0	
AGFG2	0	0.250	0	
CARMIL2	0	0.250	0	
TNFAIP2	0	0.250	0	

Supplementary Table S4

Complete VGAE-KO Validation Results (44 experiments). Percentile: (1-Rank/Total) × 100%. Bootstrap: percentage of 100 permutations where gene ranked in top 5%. Note: *RPL12* and *RPS19* were validated against *KCNA3* rather than the bridge-path-predicted *KCNA10*, as *KCNA3* showed higher expression in the validation datasets.

Hub Gene	Ion Channel	Dataset	Status	Percentile	Bootstrap	Rank
APBB1IP	*KCNA5*	SCDS0000040	Not run	—	—	—
APBB1IP	*KCNA5*	GSM5224587	Not run	—	—	—
CCDC167	*PKD2*	SCDS0000040	Not significant	69.5%	0%	—
CCDC167	*PKD2*	GSM5224587	Not significant	80.8%	0%	—
CD27	*KCNN3*	SCDS0000040	Not run	—	—	—
CD27	*KCNN3*	GSM5224587	Not run	—	—	—
CD6	*GRIK2*	SCDS0000040	Not run	—	—	—
CD6	*GRIK2*	GSM5224587	Not run	—	—	—
EXOSC5	*AQP9*	SCDS0000040	Partial (*EXOSC8*)	97.6%	100%	50
EXOSC5	*AQP9*	GSM5224587	Partial (*EXOSC8*)	98.8%	100%	24
FCRL5	*RYR3*	SCDS0000040	Not run	—	—	—
FCRL5	*RYR3*	GSM5224587	Not run	—	—	—
GALK1	*KCNA5*	SCDS0000040	Partial (*TPI1*)	99.9%	100%	3
GALK1	*KCNA5*	GSM5224587	Partial (*TPI1*)	99.6%	100%	9
ITGAL	*GRIN2A*	SCDS0000040	Not run	—	—	—
ITGAL	*GRIN2A*	GSM5224587	Not run	—	—	—
LAG3	*RYR3*	SCDS0000040	Not run	—	—	—
LAG3	*RYR3*	GSM5224587	Not significant	0.0%	0%	—
LAGE3	*ANO4*	SCDS0000040	Not significant	66.5%	0%	—
LAGE3	*ANO4*	GSM5224587	Not significant	6.0%	0%	—
LSM7	*CLIC1*	SCDS0000040	Validated	99.8%	100%	5
LSM7	*CLIC1*	GSM5224587	Validated	99.4%	100%	13
NAA10	*GRIN1*	SCDS0000040	Not significant	65.4%	0%	—
NAA10	*GRIN1*	GSM5224587	Not significant	87.8%	0%	—
PDCD5	*TRPC3*	SCDS0000040	Not significant	55.7%	0%	—
PDCD5	*TRPC3*	GSM5224587	Not significant	1.1%	0%	—
PFDN4	*CLIC2*	SCDS0000040	Not significant	15.2%	0%	—
PFDN4	*CLIC2*	GSM5224587	Not significant	1.3%	0%	—
RIPK2	*CFTR*	SCDS0000040	Partial (*HSPA8*)	99.9%	100%	2
RIPK2	*CFTR*	GSM5224587	Not significant	3.0%	0%	—
RPL12	*KCNA3*	SCDS0000040	No path gene	—	—	—
RPL12	*KCNA3*	GSM5224587	No path gene	—	—	—
RPL39	*GRIN2B*	SCDS0000040	Not significant	76.5%	0%	—
RPL39	*GRIN2B*	GSM5224587	Validated	98.5%	100%	30
RPS19	*KCNA3*	SCDS0000040	No path gene	—	—	—
RPS19	*KCNA3*	GSM5224587	No path gene	—	—	—
RPS2	*GRIN2B*	SCDS0000040	Not significant	54.2%	0%	—
RPS2	*GRIN2B*	GSM5224587	Validated	98.5%	100%	30
RPS21	*KCNQ2*	SCDS0000040	Validated	97.8%	100%	44
RPS21	*KCNQ2*	GSM5224587	Validated	98.7%	100%	26
SNRPD2	*GABRB3*	SCDS0000040	Not significant	0.1%	0%	—
SNRPD2	*GABRB3*	GSM5224587	No path gene	—	—	—
TRMT112	*CFTR*	SCDS0000040	Partial (*RPS27A*)	99.7%	100%	7
TRMT112	*CFTR*	GSM5224587	Not significant	6.4%	0%	—

Hits: number of bootstrap permutations (out of 100) where target gene ranked in top 5%.
Status categories: Validated = percentile $\geq$ 95% and bootstrap $\geq$ 95% in that dataset; Not significant = percentile < 95% or [illegible]
Partial = pathway intermediate validated but not the target ion channel itself (intermediate gene shown in parentheses);
Not run = knockout experiment not performed; No path gene = path gene not in gene set.

Supplementary Table S5

Complete Perturb-seq Validation Raw Data. Detailed results from seven complementary validation strategies for six knockout gene → ion channel pairs. All raw data values shown to enable full verification and reproducibility.

Pair / Strategy	Raw Data
LSM7→CLIC1	
**Total Evidence Score: 15.5/21**	
S1: Pseudobulk DESeq2	$p_{\text{adj}} = 0.476$, log2FC= 0.046, baseMean= 133.7
S2: GSEA Pathway	Redox_homeostasis: NES= −1.81, $p = 0.0000$, FDR= 0.0059
S3: Transcriptome Rank	Rank 1414/38606 (3.7%), $p = 0.1109$
S4: MAST Zero-Inflated	Combined $p = 0.259$, Detection: KO= 0.978 vs Ctrl= 0.985
S5: Network Mediators	8 mediators: *GPX4*, *PRDX2*, *NQO1*, *CDK4*, *CDKN1A*, +3 more
S6: Global Perturbation	Mean perturbation $z = 0.304$, 92 cells
S7: Co-expression Change	Ctrl $\rho = 0.523$, KO $\rho = 0.245$, $\Delta\rho = -0.277$
RPS21→KCNQ2	
**Total Evidence Score: 8.5/21**	
S1: Pseudobulk DESeq2	$p_{\text{adj}} = 0.608$, log2FC= −0.174, baseMean= 33.5
S2: GSEA Pathway	Potassium_channel: NES= −1.27, $p = 0.1697$, FDR= 0.3572
S3: Transcriptome Rank	Rank 3209/38606 (8.3%), $p = 0.3835$
S4: MAST Zero-Inflated	Combined $p = 0.475$, Detection: KO= 0.581 vs Ctrl= 0.608
S5: Network Mediators	1 mediator: *RPS21*
S6: Global Perturbation	Mean perturbation $z = 0.330$, 31 cells
S7: Co-expression Change	Ctrl $\rho = 0.383$, KO $\rho = 0.181$, $\Delta\rho = -0.202$
RIPK2→CFTR	
**Total Evidence Score: 7.5/21**	
S1: Pseudobulk DESeq2	$p_{\text{adj}} = 0.000$, log2FC= 5.632, baseMean= 0.0
S2: GSEA Pathway	Epithelial_transport: NES= −1.39, $p = 0.0949$, FDR= 0.1837
S3: Transcriptome Rank	Rank 20160/38606 (52.2%), $p = 0.7118$
S4: MAST Zero-Inflated	Combined $p = 1.000$, Detection: KO= 0.000 vs Ctrl= 0.002
S5: Network Mediators	1 mediator: *MUC2*
S6: Global Perturbation	Mean perturbation $z = 0.119$, 78 cells
S7: Co-expression Change	Ctrl $\rho = 0.010$, KO $\rho = 0.000$, $\Delta\rho = -0.010$
GALK1→KCNA5	
**Total Evidence Score: 6.3/21**	
S1: Pseudobulk DESeq2	Not detected (baseMean= 0)
S2: GSEA Pathway	Potassium_channel: NES= 1.75, $p = 0.0118$, FDR= 0.0178
S3: Transcriptome Rank	Rank 32175/38606 (83.3%), $p = 1.0000$
S4: MAST Zero-Inflated	Combined $p = 1.000$, Detection: KO= 0.000 vs Ctrl= 0.000
S5: Network Mediators	No mediators detected
S6: Global Perturbation	Mean perturbation $z = -0.176$, 99 cells
S7: Co-expression Change	Ctrl $\rho = 0.000$, KO $\rho = 0.000$, $\Delta\rho = 0.000$
TRMT112→CFTR	
**Total Evidence Score: 4.8/21**	
S1: Pseudobulk DESeq2	$p_{\text{adj}} = 0.000$, log2FC= 6.158, baseMean= 0.0
S2: GSEA Pathway	ABC_transporter: NES= 1.01, $p = 0.4581$, FDR= 0.9276

Continued on next page

Supplementary Table S5 (continued)

**Pair / Strategy**	**Raw Data**
S3: Transcriptome Rank	Rank 20270/38606 (52.5%), $p = 0.7440$
S4: MAST Zero-Inflated	Combined $p = 1.000$, Detection: KO= 0.000 vs Ctrl= 0.002
S5: Network Mediators	No mediators detected
S6: Global Perturbation	Mean perturbation $z = 0.610$, 61 cells
S7: Co-expression Change	Ctrl $\rho = 0.013$, KO $\rho = 0.000$, $\Delta\rho = -0.013$
EXOSC5→AQP9 **Total Evidence Score: 3.3/21**	
S1: Pseudobulk DESeq2	Not detected (baseMean= 0)
S2: GSEA Pathway	Solute_transport: NES= 1.21, $p = 0.2768$, FDR= 0.7875
S3: Transcriptome Rank	Rank 34346/38606 (89.0%), $p = 1.0000$
S4: MAST Zero-Inflated	Combined $p = 1.000$, Detection: KO= 0.000 vs Ctrl= 0.000
S5: Network Mediators	No mediators detected
S6: Global Perturbation	Mean perturbation $z = 0.098$, 84 cells
S7: Co-expression Change	Ctrl $\rho = 0.000$, KO $\rho = 0.000$, $\Delta\rho = 0.000$

Strategy descriptions:

- **S1:** Batch-aggregated pseudobulk differential expression (pyDESeq2).
- **S2:** Gene set enrichment analysis on KO-induced perturbation signature.
- **S3:** Transcriptome-wide rank of target gene in KO perturbation.
- **S4:** Zero-inflated model (MAST) accounting for dropout.
- **S5:** Indirect network mediators connecting KO to target.
- **S6:** Global perturbation magnitude (mean z-score across transcriptome).
- **S7:** Change in KO-target co-expression structure.

Supplementary Table S6

Discovery Cohort Clinicopathological Characteristics. Clinical and demographic information for the two discovery cohorts (GSE196006 and GSE251845). Age = age at surgery (years). Stage = AJCC tumor stage. Site = anatomical location of the primary tumor.

Dataset	GEO Accession	Patient ID	Sample Type	Age	Stage	Tumor Site
**GSE196006** (n=42; 21 paired tumor–normal)						
GSE196006	GSM5857798	15.018	Normal	47.8	3	Sigmoid Colon
GSE196006	GSM5857799	15.018	Tumor	47.8	3	Sigmoid Colon
GSE196006	GSM5857800	15.130	Normal	37.9	1	Ascending Colon
GSE196006	GSM5857801	15.130	Tumor	37.9	1	Ascending Colon
GSE196006	GSM5857802	15.160	Normal	47.2	3	Rectum
GSE196006	GSM5857803	15.160	Tumor	47.2	3	Rectum
GSE196006	GSM5857804	15.164	Normal	41.1	1	Rectum
GSE196006	GSM5857805	15.164	Tumor	41.1	1	Rectum
GSE196006	GSM5857806	15.171	Normal	45.5	1	Sigmoid Colon
GSE196006	GSM5857807	15.171	Tumor	45.5	1	Sigmoid Colon
GSE196006	GSM5857808	15.248	Normal	39.8	3	Cecum
GSE196006	GSM5857809	15.248	Tumor	39.8	3	Cecum
GSE196006	GSM5857810	15.288	Normal	34.9	3	Ascending Colon
GSE196006	GSM5857811	15.288	Tumor	34.9	3	Ascending Colon
GSE196006	GSM5857812	17.150	Normal	47.4	3	Rectum
GSE196006	GSM5857813	17.150	Tumor	47.4	3	Rectum
GSE196006	GSM5857814	17.477	Normal	49.8	4	Sigmoid Colon
GSE196006	GSM5857815	17.477	Tumor	49.8	4	Sigmoid Colon
GSE196006	GSM5857816	18.152	Normal	46.8	3	Sigmoid Colon
GSE196006	GSM5857817	18.152	Tumor	46.8	3	Sigmoid Colon
GSE196006	GSM5857818	18.177	Normal	39.3	3	Cecum
GSE196006	GSM5857819	18.177	Tumor	39.3	3	Cecum
GSE196006	GSM5857820	18.269	Normal	39.3	2	Descending Colon
GSE196006	GSM5857821	18.269	Tumor	39.3	2	Descending Colon
GSE196006	GSM5857822	18.466	Normal	47.3	1	Sigmoid Colon
GSE196006	GSM5857823	18.466	Tumor	47.3	1	Sigmoid Colon
GSE196006	GSM5857824	18.565	Normal	43.1	2	Sigmoid Colon
GSE196006	GSM5857825	18.565	Tumor	43.1	2	Sigmoid Colon
GSE196006	GSM5857826	19.055	Normal	39.6	3	Sigmoid Colon
GSE196006	GSM5857827	19.055	Tumor	39.6	3	Sigmoid Colon
GSE196006	GSM5857828	19.062	Normal	48.4	3	Sigmoid Colon
GSE196006	GSM5857829	19.062	Tumor	48.4	3	Sigmoid Colon
GSE196006	GSM5857830	19.076	Normal	49.2	4	Ascending Colon
GSE196006	GSM5857831	19.076	Tumor	49.2	4	Ascending Colon
GSE196006	GSM5857832	19.253	Normal	40.5	2	Ascending Colon
GSE196006	GSM5857833	19.253	Tumor	40.5	2	Ascending Colon
GSE196006	GSM5857834	20.002	Normal	49.5	1	Rectum
GSE196006	GSM5857835	20.002	Tumor	49.5	1	Rectum
GSE196006	GSM5857836	20.146	Normal	44.2	3	Rectum

Continued on next page

Dataset	GEO Accession	Patient ID	Sample Type	Age	Stage	Tumor Site
GSE196006	GSM5857837	20.146	Tumor	44.2	3	Rectum
GSE196006	GSM5857838	20.327	Normal	49.1	1	Sigmoid Colon
GSE196006	GSM5857839	20.327	Tumor	49.1	1	Sigmoid Colon
**GSE251845** (n=43; 21 tumor, 22 normal)						
GSE251845	GSM7988989	24	Tumor	52.9	2	Sigmoid
GSE251845	GSM7988990	24	Normal	52.9	–	Sigmoid
GSE251845	GSM7988991	27	Tumor	80.8	3	Cecum
GSE251845	GSM7988992	27	Normal	80.8	–	Cecum
GSE251845	GSM7988993	29	Tumor	50.8	3	Rectum
GSE251845	GSM7988994	29	Normal	50.8	–	Rectum
GSE251845	GSM7988995	30	Tumor	83.4	2	Descending colon
GSE251845	GSM7988996	30	Normal	83.4	–	Descending colon
GSE251845	GSM7988997	31	Tumor	56.1	1	Sigmoid
GSE251845	GSM7988998	31	Normal	56.1	–	Sigmoid
GSE251845	GSM7988999	32	Tumor	50.4	2	Rectum
GSE251845	GSM7989000	32	Normal	50.4	–	Rectum
GSE251845	GSM7989001	33	Tumor	63.1	4	Rectum
GSE251845	GSM7989002	33	Normal	63.1	–	Rectum
GSE251845	GSM7989003	34	Tumor	61.1	2	Sigmoid
GSE251845	GSM7989004	34	Normal	61.1	–	Sigmoid
GSE251845	GSM7989005	35	Normal	75.9	–	Sigmoid
GSE251845	GSM7989006	36	Tumor	80.8	1	Rectum
GSE251845	GSM7989007	36	Normal	80.8	–	Rectum
GSE251845	GSM7989008	37	Tumor	55.5	3	Rectosigmoid
GSE251845	GSM7989009	37	Normal	55.5	–	Rectosigmoid
GSE251845	GSM7989010	38	Tumor	80.8	3	Cecum
GSE251845	GSM7989011	38	Normal	80.8	–	Cecum
GSE251845	GSM7989012	39	Tumor	70.0	1	Rectum
GSE251845	GSM7989013	39	Normal	70.0	–	Rectum
GSE251845	GSM7989014	41	Tumor	81.0	1	Rectum
GSE251845	GSM7989015	41	Normal	81.0	–	Rectum
GSE251845	GSM7989016	42	Tumor	59.2	4	Sigmoid
GSE251845	GSM7989017	42	Normal	59.2	–	Sigmoid
GSE251845	GSM7989018	43	Tumor	50.1	1	Rectum
GSE251845	GSM7989019	43	Normal	50.1	–	Rectum
GSE251845	GSM7989020	46	Tumor	69.6	2	Sigmoid
GSE251845	GSM7989021	46	Normal	69.6	–	Sigmoid
GSE251845	GSM7989022	47	Tumor	84.0	2	Sigmoid
GSE251845	GSM7989023	47	Normal	84.0	–	Sigmoid
GSE251845	GSM7989024	49	Tumor	55.0	1	Cecum
GSE251845	GSM7989025	49	Normal	55.0	–	Cecum
GSE251845	GSM7989026	50	Tumor	68.4	4	Sigmoid
GSE251845	GSM7989027	50	Normal	68.4	–	Sigmoid
GSE251845	GSM7989028	51	Tumor	68.0	2	Hepatic flexure

Continued on next page

Supplementary Table S6 (continued)

Dataset	GEO Accession	Patient ID	Sample Type	Age	Stage	Tumor Site
GSE251845	GSM7989029	51	Normal	68.0	–	Hepatic flexure
GSE251845	GSM7989030	53	Tumor	88.8	1	Rectum
GSE251845	GSM7989031	53	Normal	88.8	–	Rectum
